# Archangelolide: A sesquiterpene lactone with immunobiological potential from *Laserpitium archangelica*

**DOI:** 10.3762/bjoc.15.189

**Published:** 2019-08-13

**Authors:** Silvie Rimpelová, Michal Jurášek, Lucie Peterková, Jiří Bejček, Vojtěch Spiwok, Miloš Majdl, Michal Jirásko, Miloš Buděšínský, Juraj Harmatha, Eva Kmoníčková, Pavel Drašar, Tomáš Ruml

**Affiliations:** 1 Department of Biochemistry and Microbiology, University of Chemistry and Technology Prague, Technická 5, 166 28, Prague 6, Czech Republic; 2 Department of Chemistry of Natural Compounds, University of Chemistry and Technology Prague, Technická 5, 166 28, Prague 6, Czech Republic; 3 Charles University in Prague, Faculty of Medicine in Pilsen, 301 66 Pilsen, Czech Republic; 4 Institute of Organic Chemistry and Biochemistry, Academy of Sciences of the Czech Republic, Flemingovo náměstí 2, 166 10 Prague 6, Czech Republic; 5 Institute of Experimental Medicine, Academy of Sciences of the Czech Republic, v.v.i., 14220 Prague 4, Czech Republic

**Keywords:** anti-inflammatory properties, archangelolide, dansyl fluorescent conjugate, sarco/endoplasmic reticulum calcium ATPase, sesquiterpene lactone, trilobolide analogue

## Abstract

Sesquiterpene lactones are secondary plant metabolites with sundry biological effects. In plants, they are synthesized, among others, for pesticidal and antimicrobial effects. Two such compounds, archangelolide and trilobolide of the guaianolide type, are structurally similar to the well-known and clinically tested lactone thapsigargin. While trilobolide has already been studied by us and others, there are only scarce reports on the biological activity of archangelolide. Here we present the preparation of its fluorescent derivative based on a dansyl moiety using azide–alkyne Huisgen cycloaddition having obtained the two sesquiterpene lactones from the seeds of *Laserpitium archangelica* Wulfen using supercritical CO_2_ extraction. We show that dansyl-archangelolide localizes in the endoplasmic reticulum of living cells similarly to trilobolide; localization in mitochondria was also detected. This led us to a more detailed study of the anticancer potential of archangelolide. Interestingly, we found that neither archangelolide nor its dansyl conjugate did exhibit cytotoxic effects in contrast to the structurally closely related counterparts trilobolide and thapsigargin. We explain this observation by a molecular dynamics simulation, in which, in contrast to trilobolide, archangelolide did not bind into the sarco/endoplasmic reticular calcium ATPase cavity utilized by thapsigargin. Last, but not least, archangelolide exhibited anti-inflammatory activity, which makes it promising compound for medicinal purposes.

## Introduction

**Figure 1:**
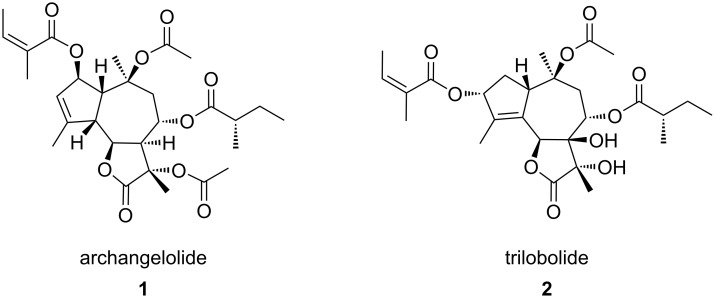
The structure of the sesquiterpene lactones archangelolide (**1**) and trilobolide (**2**).

Sesquiterpene lactones (SLs) have been attracting interest already for some time due to the plethora of biological effects they elicit. Various SLs show anticancer, antimicrobial, antioxidant, antiprotozoal, antiviral and immunobiological activities (reviewed in [1–2]). Two SLs remarkable for their immunobiological potential are archangelolide (**1**) and trilobolide (**2**), depicted in Figure 1. Compound **2** exhibits strong induction of nitric oxide (NO) in eukaryotic cells, which in turn evokes the synthesis of IL-6, INF-γ and TNF-α (see section “Abbreviations” at the end of the text). Furthermore, compound **2** has a very similar structure to the well-described SL thapsigargin, which is the best-known inhibitor of sarcoplasmic/endoplasmic reticular calcium ATPase (SERCA) with *K*_i_ values in the range of nanomoles [3]. The inhibition of SERCA by thapsigargin is stoichiometric and irreversible [4] and results in the depletion of the intracellular calcium storage and an elevated cytosolic calcium concentration, which then can trigger apoptosis in cells. This ability of thapsigargin to trigger programmed cell death prompted the development of mipsagargin/G202 [5], a thapsigargin-derived prodrug that completed phase-I and phase-II clinical trials [6] for several types of solid tumors. The structurally similar compound **2** was found to act by the same mechanism in cells as thapsigargin [7] and was also found to be cytotoxic with localization to the endoplasmic reticulum [8]. The cytotoxicity of compound **2** prompted the preparation and evaluation of compound **2** conjugates with porphyrins [9] and steroids [10] for targeted uptake of compound **2** into cancer cells, and the conjugates showed interesting biological effects including antimycobacterial effects [10]. Nevertheless, cytotoxicity posed a considerable limitation for the utilization of compound **2**. It was, however, soon found that compound **2** may be modified in such a way that its cytotoxicity is reduced while the immunobiological properties are retained [11].

Contrary to compound **2**, compound **1** inhibits NO production and synthesis of IL-1β and INF-γ [12]. Although its structure was already elucidated in the 1970s [13], the information on cytotoxicity of compound **1** is very limited [12] and its intracellular localization and mechanism of action are unknown. Therefore, based on the structural similarity between compound **1** and **2**, we suspected that new interesting and relevant information on this biologically largely undescribed SL could be identified.

In order to study the action of these commercially unavailable compounds, efficient isolation must first be performed in order to gain sufficient amount of pure SLs. Even though the presence of several immuno-active SLs, including compound **1** and **2**, was previously reported in *Laser trilobum* (L.) Borkh and the method of their isolation from petroleum ether extracts of plant roots [12] was described, we aimed to develop a facile method for the isolation of these SLs from the seeds of *Laserpitium archangelica* Wulfen using supercritical CO_2_ extraction (SCE; for advantages of this method, see Supporting Information File 1, section 8). In our experience, any part of the plant may be used for extraction of compound **1**, while the roots and seeds give much higher yields than other parts. Utilization of SCE gives improved efficiency of the extraction and shortens the work-up in contrast with classical methods.

By the abovementioned methodology, we obtained sufficient amounts of these compounds for further work including synthetic modifications using azide–alkyne Huisgen cycloaddition. We previously showed the preparation of fluorescent trilobolide conjugates [8] that retained the activity of the parental compounds and proved to be useful for live-cell imaging. In this article, we present a similar approach for compound **1**. Using cancerous, non-transformed, and, also primary cell lines, we evaluated the viability of cells treated with compound **1** and its fluorescent dansyl conjugate, which we then used to study its intracellular localization. Finally, we also determined the anti-inflammatory activity of compound **1** using rat macrophages.

## Results and Discussion

### *L. archangelica* metabolite isolation and identification

For isolation of the major metabolites of *L. archangelica,* we used 100 g of fine ground seeds (Supporting Information File 1, Figure S2) and the method of supercritical CO_2_ extraction, which was carried out under pressure of 40 MPa at 40 °C until the extract substances passed from the extracted material (when several subsequent fractions were not adding any addition to the weight to the extract). The obtained yellowish extract (42.5 g) was stored in fridge for a period of 72 h during which the crystals were formed. These were collected by filtration over a frit and washed with hexanes obtaining 7.9 g of the matter that was analyzed by TLC and LRMS analyses. We observed that the matter consisted, in particular, of two terpene-type compounds, compound **1** and **2**, and of β-sitosterol. Using TLC, we found that the mother liquor contained minimal quantities of compound **1** and **2**, but was abundant in β-sitosterol. Therefore, the obtained matter was further re-dissolved in methylene chloride and coated on silica. The purification by column chromatography using a toluene–diethyl ether gradient system (0%→10% of ether) yielded 2.7 and 1.2 g of compound **1** and **2**, respectively. The identity of these two SLs was verified by NMR, HRESIMS and IR analyses (see Supporting Information, section 1). The melting points of the compounds were also in accordance with previously reported data. Compound **1** was crystalized from a mixture of diethyl ether/hexanes (mp 108–111 °C) [14] and compound **2** from a mixture of toluene/Et_2_O (mp 190–192 °C) [12].

### Derivatization of compound **1** with a dansyl probe

In order to evaluate the intracellular localization and the fate of compound **1** in living cells, we synthesized a blue-emitting derivative employing a dansyl label. This fluorophore is convenient not only for its small size but also for its membrane permeability. Moreover, it was successfully used in other studies of visualizing other natural compounds [15–17].

**Scheme 1:**
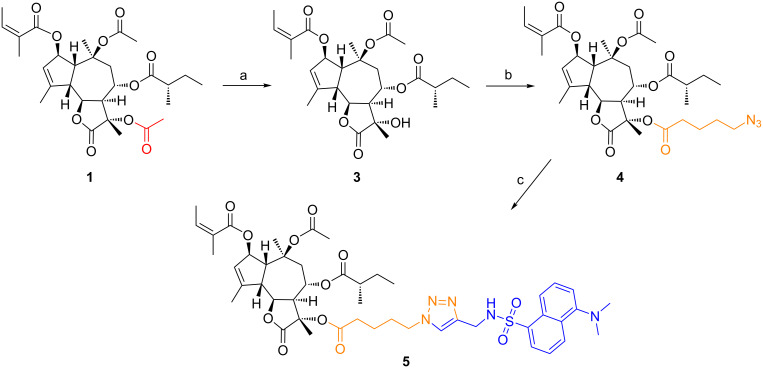
Reagents and conditions: a) MeOH, TEA, 48 h, yield 32%; b) (i) 5-azidopentanoic acid, DCC, DCM, 90 min, rt; (ii) 4-DMAP, DCM, 8 h, rt, 86%; c) dansylated propargylamine, CuI, TBTA, THF, MW, 70 °C, 2 h, yield 64%.

The synthesis (Scheme 1) of the dansyl-labeled archangelolide **5** started with a mild solvolysis of compound **1** by triethylamine in methanol. Surprisingly, the only product that we obtained after 48 h of treatment was 11-deacetylarchangelolide (**3**), in only 32% yield. The position in the structure of compound **1** where deacylation takes place remarkably differs from that in compound **2** and thapsigargin. This is very likely due to stereochemistry on the lactone ring connection at C7 and the absence of a hydroxy group at the same position. The steric circumstances and the presence of the α-carbonyl group makes the tertiary C11 hydroxy group more acidic and thus a better leaving group in the reaction. Next, the quaternary 11α-hydroxy group was acylated by freshly prepared 5-azidopentanoic anhydride in the presence of 4-DMAP at room temperature. Finally, the azidopentanoate **4** was successively introduced into a click reaction with fluorescent 5-(dimethylamino)-*N*-(prop-2-yn-1-yl)naphthalene-1-sulfonamide (synthesized according to [18]) using CuI and TBTA [19] catalysis. All of the synthesized compounds were thoroughly described by NMR (Supporting Information File 1, Table S1), IR, optical rotation (Supporting Information File 1, section 2) and HRMS (Supporting Information File 1, section 3). All compounds were re-purified on a short silica column prior to testing and afterwards lyophilized from *tert*-butanol*.* The substances were analyzed by HPLC proving a purity of ≥95% (Supporting Information File 1, section 4). Fluorescent properties of compound **5** were determined in PBS and methanol, the emission maxima corresponded to 484 and 519 nm in these two solvents, respectively (Supporting Information File 1, Figure S17).

### Intracellular localization of compound **5**

**Figure 2:**
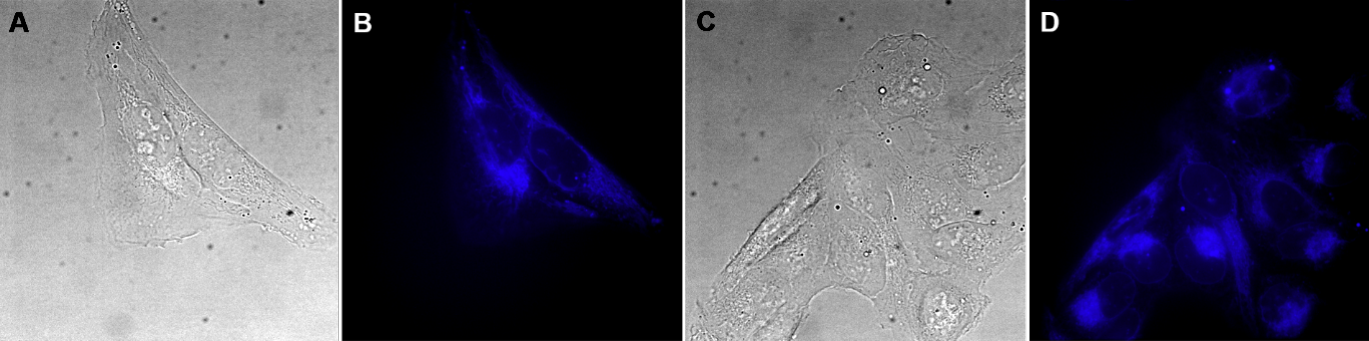
Intracellular localization of archangelolide-dansyl (**5**) in human cells from osteosarcoma (U-2 OS). A, C) Bright-field images; B, D) fluorescence microscopy of living cells treated with 1 µM concentration of compound **5** for 90 min.

**Figure 3:**
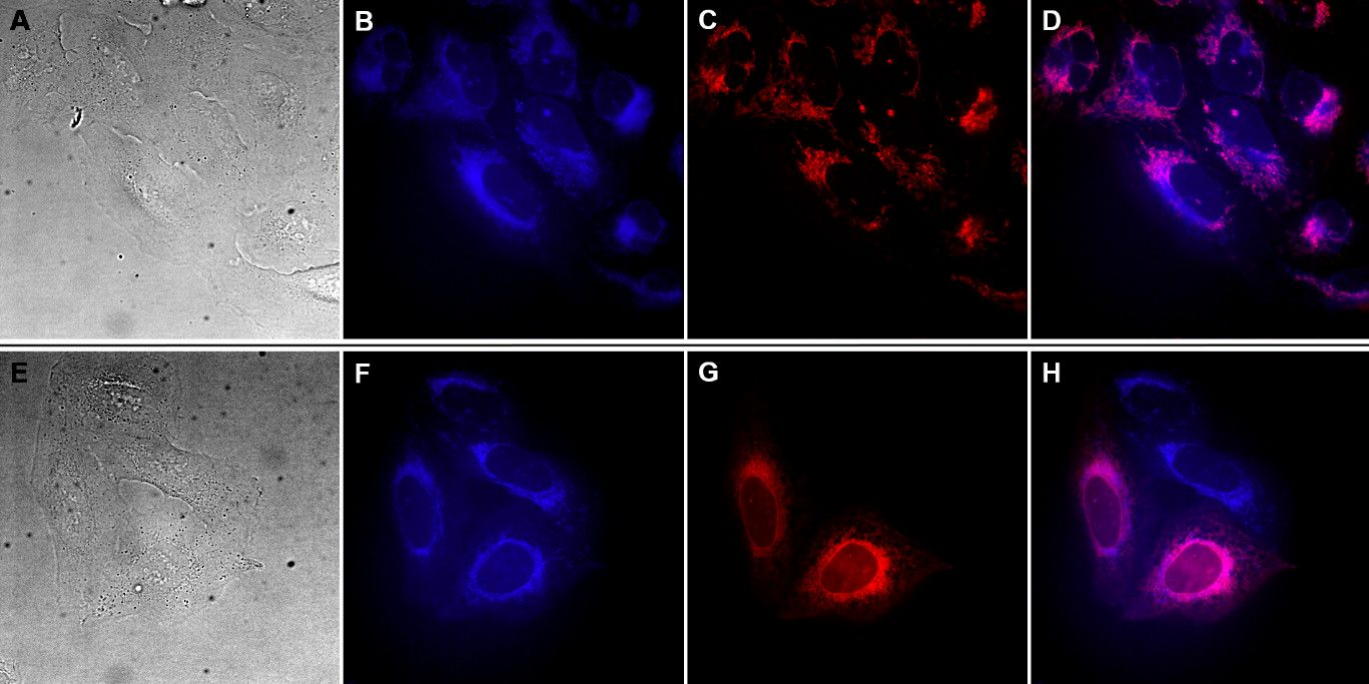
Co-localization of dansylarchangelolide **5** with a marker of endoplasmic reticulum (top row) and with a mitochondrial marker (bottom row) in human cells from osteosarcoma (U-2 OS). A, E) Bright-field images. Fluorescence microscopy of living cells treated with 1 µM concentration of compound **5** (90 min; images B and F) and a mitochondria-specific dye from [20] (10 min; image C) or pDNA coding mCherry-ER (image G). D, H) merged images.

In this study we aimed to evaluate the biological effects of the poorly described natural compound archangelolide (**1**) and its fluorescent dansyl conjugate **5** in primary, cancer and primary immune cells. First, we analyzed the rate of cell uptake of compound **5** and dansyl amide in U-2 OS (Figure 2 and Supporting Information File 1, Figure S18) and MRC-5 (Supporting Information File 1, Figure S19) cells. In both cell lines, compound **5** localized already after 30 min at 0.5 µM concentration, and the fluorescence intensity increased over a period of 2 h of incubation. In order to identify the intracellular localization of compound **5**, co-localization experiments using endoplasmic reticulum and mitochondrial markers were performed. As it is apparent from Figure 3 and Figure S20 (Supporting Information File 1), compound **5** localized in the endoplasmic reticulum, which is in agreement with the site of localization of another SL, the SERCA inhibitor compound **2** [8]. However, we observed partial co-localization also in mitochondria, in which SERCA is not present. Localization of a control (dansyl fluorophore) was not organelle-specific and the fluorescence intensity was very weak even at 2 µM concentration (Supporting Information File 1, Figure S18).

### Impact of compound **1** on cell viability

In order to reveal whether compound **1** is as potent as thapsigargin or compound **2** in terms of inhibition of cancer cell proliferation, we evaluated its cytotoxicity in a number of cell lines originating from various tumors: prostate, osteosarcoma, breast, colon, pancreas and lung. Cells were treated with compound **1** and its derivatives up to a final concentration of 50 µM for periods of 24, 48 and 72 h. Surprisingly, compound **1** exhibited almost no cytotoxic effect under these conditions (see Supporting Information File 1, Table S2 and Table S3, Figures S21–S23). The IC_50_ values were in the upper micromolar range and up to the tested concentration of 50 µM, 50% cell death was only reached for HEK 293T and LNCaP after 72 h of treatment. Moreover, we also did not detect toxicity of compound **1** in primary and transformed cell lines. Based on these differences from thapsigargin and compound **2**, it seems that this SL does not act as SERCA inhibitor. Therefore, we proceeded to confirm this hypothesis by a molecular dynamics simulation study.

### Molecular dynamics simulation of compounds **1** and **2** with SERCA

The binding cavity for thapsigargin and compound **2** in the SERCA protein lies in the transmembrane domain between the helices 3, 5 and 7 [7,21]. Comparing the polarity of these compounds with compound **1** based on the formation of hydrogen bonds, the most polar compound capable of forming the highest number of hydrogen bonds is thapsigargin, the least polar is compound **1** (for details, see Supporting Information File 1, Table S4). Nevertheless, the complex of SERCA with thapsigargin is preferentially stabilized by hydrophobic interactions, [21] and all three mentioned SLs are strongly hydrophobic molecules. We therefore assumed that compounds **1** and **2**, which can form even fewer hydrogen bonds than thapsigargin, may interact with SERCA similarly to thapsigargin. However, this is clearly not the case as represented by our cytotoxicity evaluation using multiple cell lines.

In order to elucidate why compound **1** does not exhibit the same cytotoxicity as its structural counterparts thapsigargin and compound **2**, the potent SERCA inhibitors, manual docking of compound **1** into SERCA followed by MD simulation (1–10 ns) was performed. For this simulation, four different complexes of SERCA and SL were chosen: i) compound **1** positioned correspondingly to the orientation of DTB in the SERCA binding cavity (simulation 1); ii) compound **1** rotated by 180° (simulation 2); iii) compound **2** (for comparison) used as a ligand with the orientation equal to DTB (simulation 3); iv) SERCA positioned in a phospholipid membrane to reflect the fact that it is a transmembrane protein, with compound **2** as a ligand (simulation 4).

The first round of simulations (1–10 ns) of compound **1** docking into SERCA cavity with compound **1** constrained as described (simulation 1) showed the presence of hydrophobic interactions of ligand side chains with Phe256, Val263, Ile829 and the hydrophobic part of Gln259 amino acid residues. Gln259 was the main residue involved in hydrophilic interaction. Interestingly, during the simulations in water environment, compound **1** had the tendency to escape from the SERCA cavity.

In simulation 2 of compound **1** rotated by 180° with constrained positions (10 ns), interactions with Phe256 and hydrophobic parts of Glu255 and Gln259 were detected. When the simulation was performed without constrained positions, compound **1** escaped from the SERCA protein cavity.

**Figure 4:**
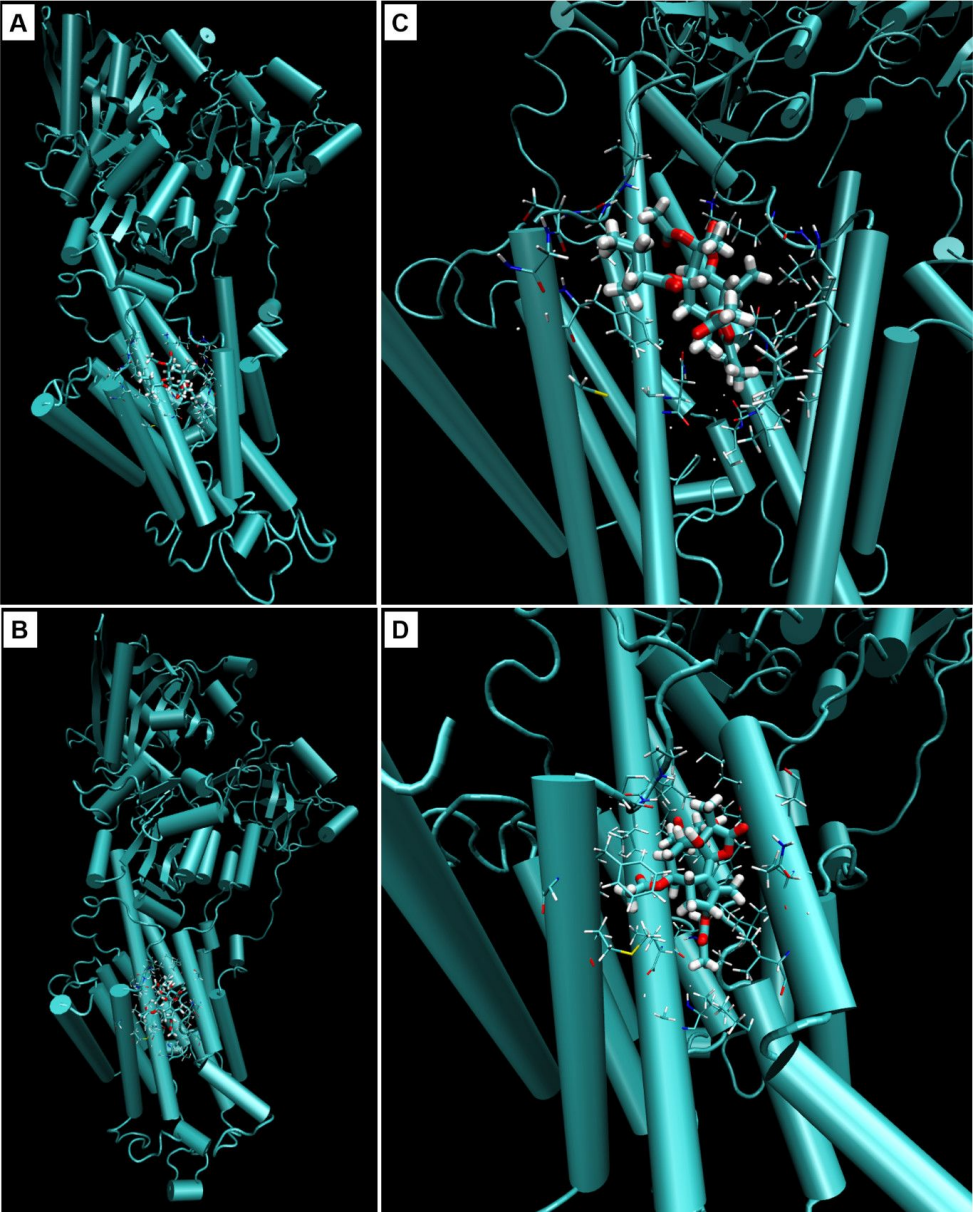
Cartoon representation of sarco/endoplasmic reticulum Ca^2+^ ATPase binding pocket with A, C) archangelolide (**1**) or B, D) trilobolide (**2**) after molecular dynamic simulations. Depicted are also amino acid residues in a range of 5 Å from the respective ligand. The images were created using VMD software, version 1.9.2.

**Figure 5:**
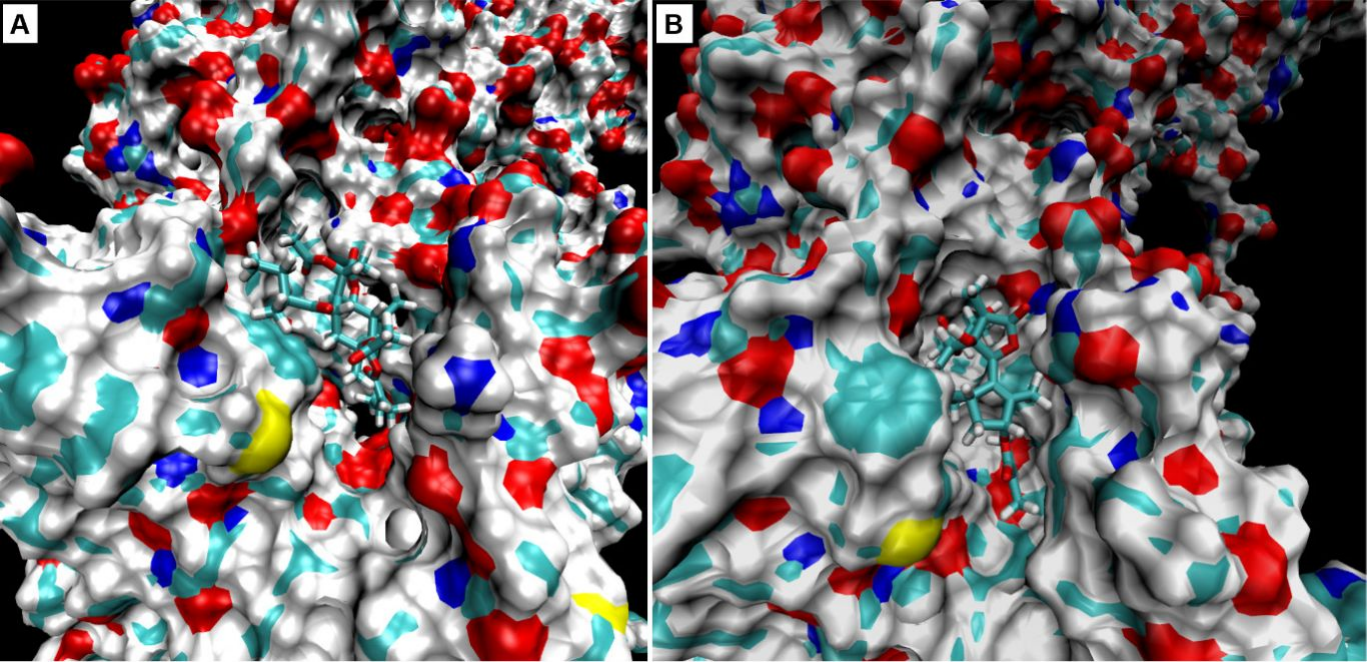
Molecular surface representation of sarco/endoplasmic reticulum Ca^2+^ ATPase binding pocket with A) archangelolide (**1**) and B) trilobolide (**2**) after molecular dynamic simulations. SURF function and probe size 1.4 Å were used. SURF function was written by Amitabh Varshney in University of North Carolina. The images were created using VMD software, version 1.9.2.

After the first simulation of compound **2** (simulation 3) in non-water environment, hydrogen bonds of compound **2** with Glu255 and Gln259 residues, and α-amino group of Ile829 were obvious. The hydrophobic interactions were directed preferentially to Phe256, Val263, Leu260 and Ile829 residues and a hydrophobic part of Lys252. In contrast to simulation 1 with compound **1**, there was no indication of compound **2** escaping from the SERCA cavity (Figure 4 and Figure 5).

During simulations with SERCA positioned in a phospholipid membrane (simulation 4; 1 ns), the free space between individual phospholipids in the cell membrane was filled. SERCA remained stable and tightly embedded in the membrane and did not show any tendency to escape from it. Compound **2** interacted similarly to simulation 3, in which it remained in the thapsigargin SERCA cavity for the duration of the simulation.

**Figure 6:**
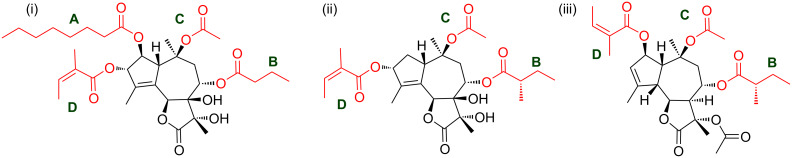
Structural formulae of (i) thapsigargin, (ii) trilobolide (**2**), and (iii) archangelolide (**1**). Red parts show structural moieties of thapsigargin and its derivatives contributing to SERCA binding affinity (according to [22]): A) octanoyl, B) butanoyl or 2-methylbutanoyl, C) acetyl, D) angeloyl.

When compound **1** was positioned in the same way as compound **2** and DTB during the simulations, it showed a tendency to escape from the SERCA cavity, which was significantly pronounced in the case of compound **1** rotated by 180°. Therefore, we are convinced that especially the orientation and positions of the side chains of compound **1** are very important for its affinity to SERCA or the lack thereof. Winther and co-workers [22] reported that the dissociation constant of thapsigargin derivatives each lacking one of the four thapsigargin side chains (e.g., 2-deoctanoyl-4,5-dihydrothapsigargin or 8-*O*-(dodecanoyl-8-*O*-debutanoyltrilobolide) increases in the following order: *O*-2-deoctanoyl < *O*-8-debutanoyl < *O*-10-deacetyl < *O*-3-deangeloyl. The individual side chains are depicted in Figure 6.

Considering the inhibition potency of SERCA by thapsigargin and compound **2** [7,23] we are not convinced that the missing structural moiety *O*-2-octanoyl (see Figure 6, A) in the case of compound **1** and **2** has a significant influence on the affinity to SERCA. Furthermore, the *O*-8 butanoyl and *O*-10 acetyl moieties (Figure 6, B and C) are not significantly different in compound **1** (presence of a methyl group in the *O*-8 butanoyl side chain) from those of thapsigargin and compound **2**. In contrast, the *O*-3 angeloyl moiety (Figure 6, D) is positioned at C3 in thapsigargin and compound **2**, whereas in compound **1** it is at C2. An *O*-3 deangeloylthapsigargin derivative was previously prepared by Winther and co-workers, who reported that 500–600 times higher concentrations of this derivative than of thapsigargin were needed to achieve 50% inhibition of SERCA, which was the highest of all the derivatives lacking a single side chain [22]. Taking into consideration their result as well as our simulations, we found that compound **1** is unlikely to act as a SERCA inhibitor and, also, it seems that the *O*-3 angeloyl moiety might play a rather significant role in the affinity of thapsigargin/compound **2** to SERCA.

### Immunobiological properties of compound **1**

SLs, natural compounds predominantly isolated from the species *Asteraceae* and *Apiaceae* represent a rich source of small molecules with potential pharmacological effects. Indeed, some of them have reached clinical applications as antimalarial (artemisinin; [24]) or antitumor (thapsigargin; [25]) agents or have been experimentally investigated with an increasing rate (see PubMed results by year).

**Figure 7:**
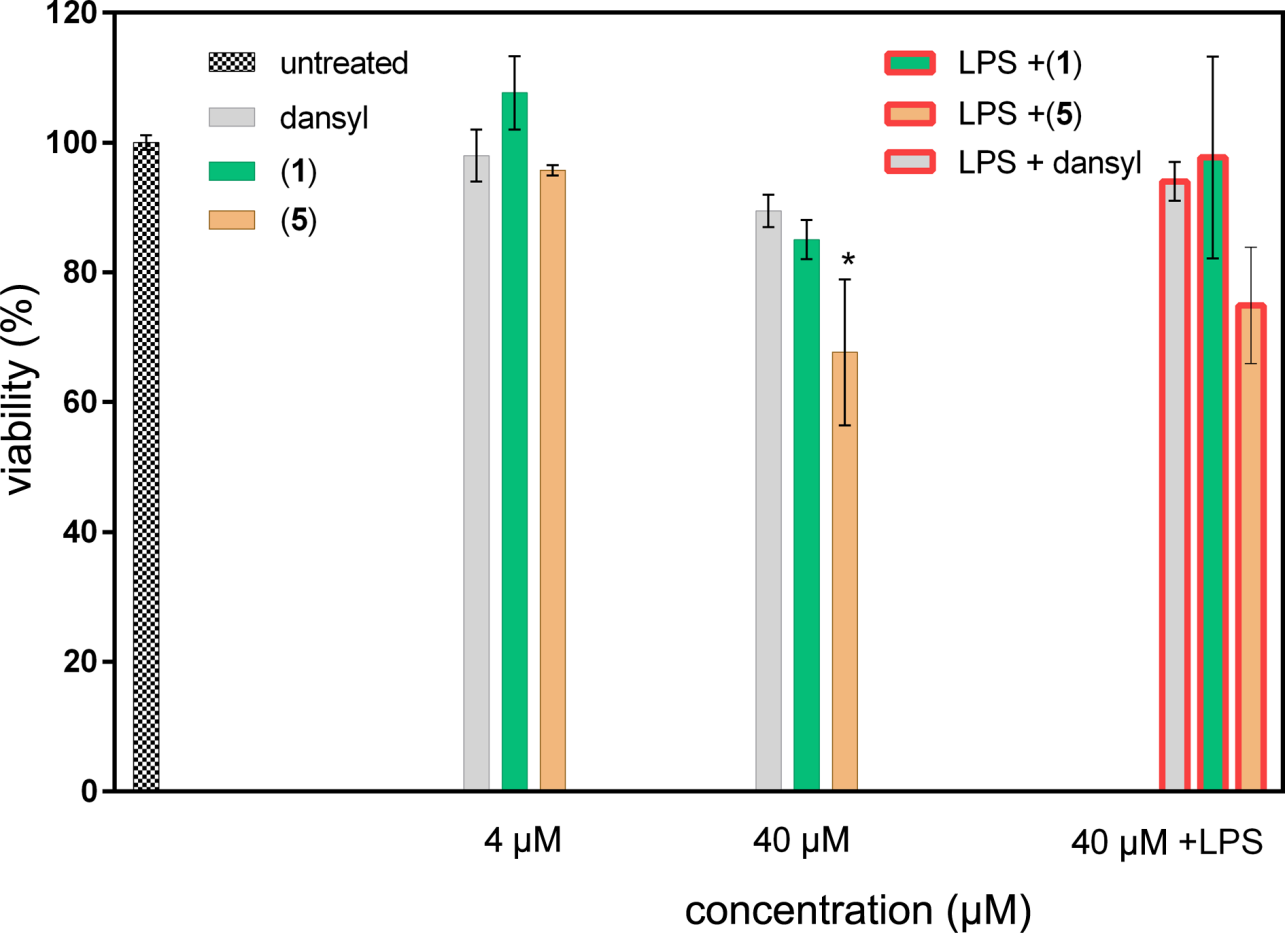
Viability of rat peritoneal cells treated with archangelolide (**1**), dansylarchangelolide **5** and dansyl amide itself. Compounds were applied at 4 µM and 40 µM concentrations and cells were cultured for 24 h. WST-1 assay was used for viability evaluation. The results are expressed as percentage of untreated control ± SEM of *n* = 6–8 values from two independent experiments. Statistical significance: *P < 0.05, the results of compound **5** are statistically different from those of untreated cells.

In order to assess the immunobiological potential of compound **1**, its cytotoxicity had to be determined first. Therefore, we measured its impact on the viability of rat peritoneal macrophages after 24 h of treatment. No cytotoxicity was detected at tested concentrations of 4 and 40 µM (Figure 7). This is in accordance with results of [12], in which compound **1** and 2-deangeoylarchangelolide did not exhibit any cytotoxic effect. On the other hand, compound **5** partially decreased the viability of rat peritoneal macrophages to 67% (*P < 0.05) at 40 µM concentration. It seems that this phenomenon was not related to the fluorescent dye of compound **5**, since dansyl amide itself did not change the viability of rat macrophages at both concentrations. A decrease in cell viability to 75% (statistically not significant) was also recorded when rat primary macrophages were incubated with 1000 pg·mL^−1^ LPS + 40 µM compound **5**. However, no toxicity was observed for the combination of LPS + dansyl amide at the same concentration.

**Figure 8:**
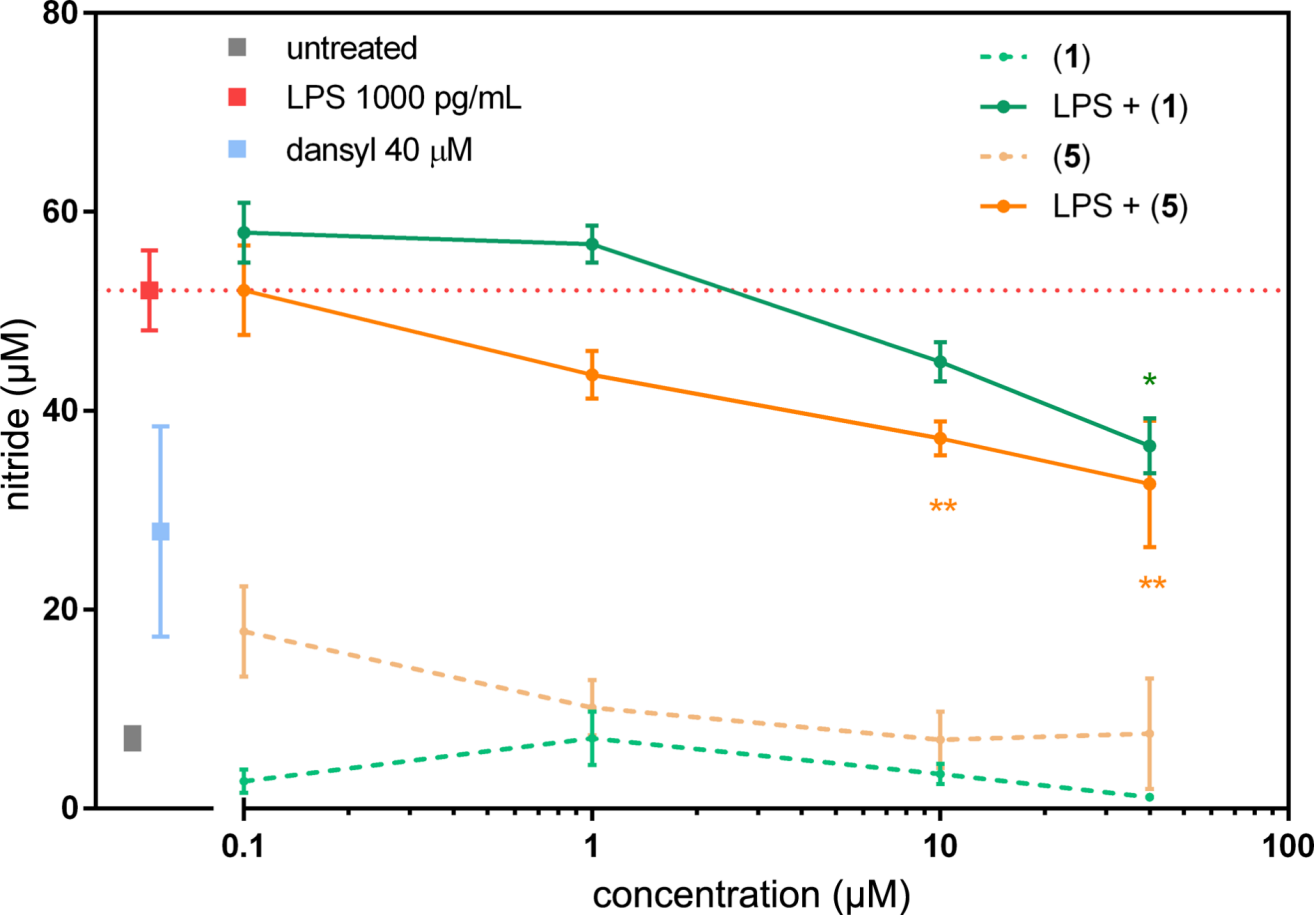
NO production in primary rat macrophages. The cells were treated with archangelolide (**1**) and dansylarchangelolide **5** in the concentration range of 0.1–40 µM for 24 h with or without lipopolysaccharide (LPS, 1000 pg·mL^−1^) or with solely 40 µM dansyl amide. The results represent the mean ± SEM of three independent experiments, *n* = 6. Statistical significance: * *P* < 0.01, **P < 0.001, the results of the compounds are statistically different from those of the LPS-treated cells.

Immunomodulatory [26] and anti-inflammatory [27] effects of many SLs are well documented. In this study, we investigated the ability of compound **1** and **5** to modulate NO and cytokine TNF-α. We found that when rat primary macrophages were stimulated with 1000 pg·mL^−1^ of LPS, a dose-dependent decrease in NO production was present in cells treated with compound **1** (Figure 8). The decrease in NO was significant (*P < 0.01) for 40 µM concentration of compound **1**. The inhibitory effect on NO production was more pronounced in cells treated by compound **5**, with statistics **P < 0.001 for both concentrations, 10 and 40 µM. It is possible that the decline in NO at 40 µM concentration of compound **5** was at least partially induced by reduced cell viability. On the other hand, dansyl amide alone did not lower NO production.

In contrast to other SLs such as thapsigargin [28] or compound **2** [29] and its derivatives [9], compound **1** did not exhibit immunostimulatory activity, which was confirmed in this study. On the other hand, a slight to moderate effect on the decrease in NO production is clear and corresponds to the result of [12]. Thus, we show the biological activity of a semi-synthetic conjugate of compound **1** as inhibitor of NO production at very low micromolar concentrations.

**Figure 9:**
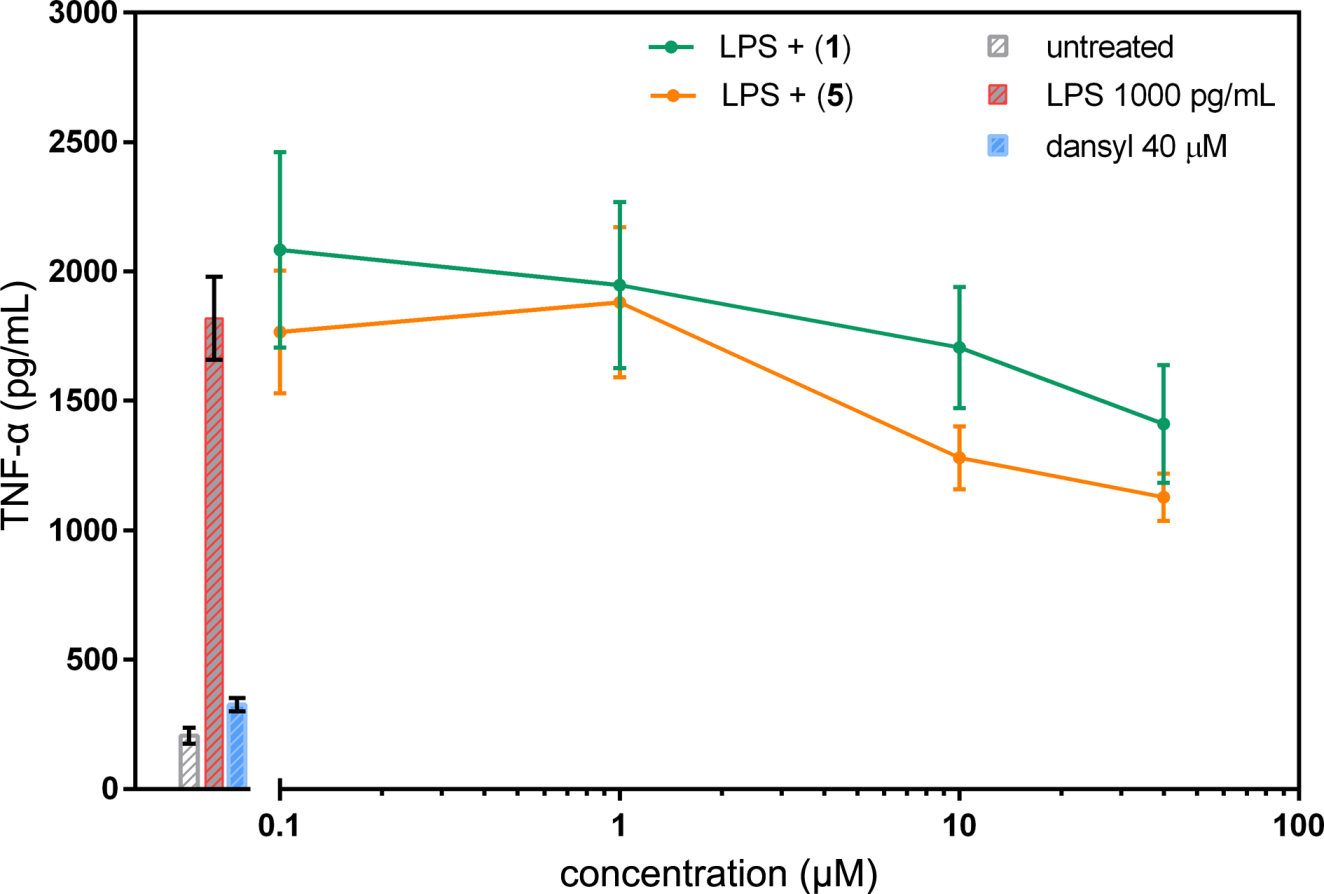
Evaluation of cytokine TNF-α secretion in rat peritoneal cells. Stimulation of primary cells was induced by 1000 pg·mL^−1^ of LPS. Cells were cultured in the presence of archangelolide (**1**) and dansylarchangelolide **5** for 24 h. Cytokine secretion was detected by ELISA. The data are the means ± SEM of two independent experiments, *n* = 4.

**Figure 10:**
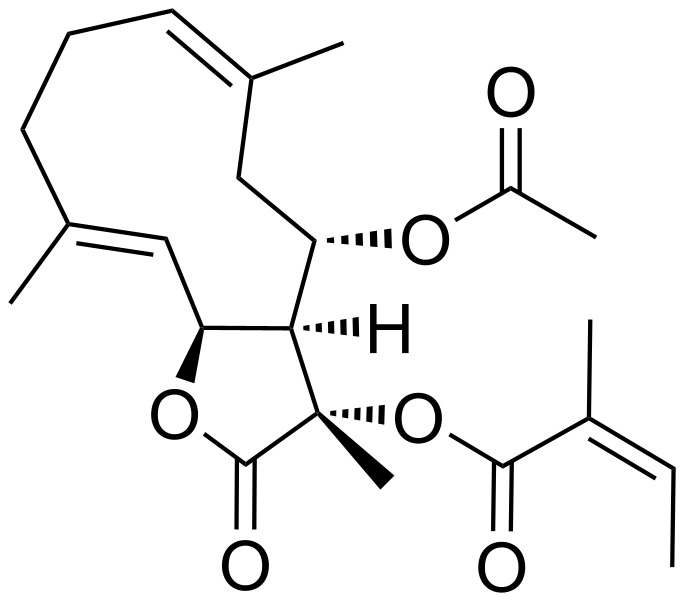
Structure of laserolide.

Furthermore, the anti-inflammatory activity of compounds, which is usually demonstrated by inhibitory activity against TNF-α secretion, has never been explored for compound **1**. Therefore, in this study, we examined the effect of compound **1** and **5** on TNF-α secretion in rat primary macrophages (Figure 9). We found a mild decrease in TNF-α secretion in LPS-treated cells for both compounds. We did not detect any effect of dansyl amide or dansyl amide + LPS (data not shown) on cytokine levels. Previously, weak anti-inflammatory activity was also found for cytokine IL-6 and negligible inhibition of Il-1β was detected for compound **1** and 2-deangeolylarchangelolide [12], whereas a strong anti-inflammatory effect was found for another SL, laserolide [12]. Laserolide (Figure 10) is a germacrane-type SL that has a ten-membered ring adjoining the five-membered lactone ring whereas compound **1** belongs to the guaiane type. In guaianolides, a bond is present between C1 and C5 of the ten-membered ring, creating a seven-membered and a five-membered ring. Nevertheless, the arrangement of the five-membered lactone ring to the ten-membered part of the molecule with regards to adjoining substituents is similar in both compound **1** and laserolide. This implies that the arrangement of the five-membered ring in a SL molecule towards the rest of the molecule may be related to its anti-inflammatory activity.

## Conclusion

Having developed a method for the isolation of two related SLs, archangelolide (**1**) and trilobolide (**2**), from the seeds of *Laserpitium archangelica* Wulfen, we extended the knowledge on the first one, so far almost undescribed. We revealed that despite a high degree of structural similarity with two cytotoxic SLs, compound **2** and thapsigargin, compound **1** exhibited cytotoxicity neither in the tested cancer cell lines nor in primary cells. To explain the reason for different cytotoxicity of compound **1** compared to compound **2** and thapsigargin, we prepared its fluorescent derivative for live-cell imaging studies, which determined its intracellular localization in endoplasmic reticulum and mitochondria of both cancer and normal cells. This led us to examine the binding of compound **1** to SERCA, the target of compound **2** and thapsigargin, by a docking study. Indeed, the results strongly argue that compound **1** is biologically very distinct from the other two SLs, since it does not bind and probably also not inhibit SERCA. Finally, compound **1** exhibited anti-inflammatory activity, which was demonstrated by a decrease in NO production and TNF-α secretion in rat primary macrophages. This makes compound **1** an interesting SL for further study of its immunobiological activity.

## Experimental

### Source of the natural material

Fresh seeds of *Laserpitium archangelica* Wulfen were provided by the Farmacognostic garden in Poznan (Mazowiecka Street) of the Medical University in Poznan (Poland). The plant was identified by a research specialists of the Institute of Organic Chemistry and Biochemistry, Academy of Sciences of the Czech Republic. A voucher specimen has also been deposited at the Department of Natural Compounds at the Faculty of Food and Biochemical Technology, University of Chemistry and Technology, Prague (CZ). Compound **2** was obtained from SciTech spol. s r. o. Prague (CZ).

### Synthesis

The synthesis is depicted in Scheme 1.

#### 11-Deacetylarchangelolide (**3**)

Compound **1** (500 mg, 0.91 mmol) was dissolved in freshly distilled MeOH (10 mL) and TEA (2.5 mL) was added dropwise over 1 h period. The mixture was stirred for 48 h at room temperature (ca. 23 °C); then, the solvents were removed under reduced pressure and the product was chromatographed twice (hexanes/AcOEt, gradient 5:1→3:1) to obtain 11-deacetylarchangelolide (148 mg, 0.29 mmol) in 32% yield. Unreacted compound **1** (256 mg) was recovered. The yield based on recovered starting material was 66%. *R*_f_(**1**) = 0.6; *R*_f_(**3**) = 0.4 in hexanes/AcOEt, 3:1. For analytical data, see Supporting Information File 1, sections 2–4.

#### Synthesis of 11-azidovaleroyl-11-deacetylarchangelolide (**4**)

5-Azidopentanoic acid (80 mg, 0.56 mmol) was dissolved in dry CH_2_Cl_2_ (3 mL), then DCC (58 mg, 0.28 mmol) was added and the mixture was stirred for 90 min at room temperature (ca. 23 °C) after which the DCU was filtered out. 11α-Deacetylarchangelolide (60 mg, 0.12 mmol) and 4-DMAP (18 mg, 0.14 mmol) were dissolved in CH_2_Cl_2_ (3 mL) and freshly prepared anhydride of 5-azidopentanoic acid was added to the stirred solution. The mixture was stirred at room temperature for 8 h, after which it was filtered and the solvents were removed under reduced pressure. The residue was chromatographed twice (toluene/Et_2_O, 10:1) to obtain the final product (66 mg, 0.1 mmol) in 86% yield with *R*_f_ = 0.75 in hexanes/AcOEt, 3:1. For analytical data, see Supporting Information File 1, sections 2–4.

#### Synthesis of 11-(5-(4-((5-(dimethylamino)naphthalene-1-sulfonamido)methyl)-1*H*-1,2,3-triazol-1-yl)pentanoylarchangelolide (**5**)

To a solution of azidovalerate **4** (40 mg, 0.063 mmol) and 5-(dimethylamino)-*N*-(prop-2-yn-1-yl)naphthalene-1-sulfonamide (20 mg, 0.07 mmol) in THF (3 mL), CuI (50 µL of 1 M water solution) and TBTA (3 mg, 5.6 µmol) were added. The mixture was placed into a microwave reactor and irradiated for 2 h at 70 °C. Then, the solvent was evaporated under reduced pressure and the residue was chromatographed (hexanes/AcOEt, 1:1). The obtained product **5** was re-chromatographed twice to obtain pure product (37 mg, 0.04 mmol) as a slightly yellowish solid in 64% yield with *R*_f_ = 0.25 in hexanes/AcOEt, 1:1. For analytical data, see Supporting Information File 1, sections 2–4.

### Computational studies

#### MD Simulations of compound **1** and **2** with sarco/endoplasmic reticular calcium ATPase

For docking and following simulations, a structure of sarco/endoplasmic reticular calcium ATPase (SERCA) protein bound to 8-*O*-(dodecanoyl-8-*O*-debutanoyltrilobolide) [DTB] with 3NAL code was obtained from Protein Data Bank [22]. The structure of compound **1** was drawn using MarvinSketch 6.006 software (ChemAxon Ltd.), the structure of compound **2** was obtained by editing the DTB structure.

First, we computed General Amber Force Field parameters for molecules of compound **1** and **2** in Antechamber software. Charges were calculated by restrained electrostatic potential method (RESP) based on a wave function calculated using quantum chemistry at the HF/6-31G*//HF/6-31G* level. Compound **1** molecule was manually docked into the SERCA binding site for thapsigargin using UCSF CHIMERA 1.10.2 (University of California, San Francisco) software by two approaches. In the first one, the molecule was docked so that the spatial orientation of the central seven-membered ring reached the best agreement with the orientation of DTB. In the other approach, the docked molecule was rotated over by 180°. The following simulations proceeded with the same parameters independently of each other.

The computed complex was transformed into a box with the size of 10.309 × 10.111 × 15.063 nm^3^ with periodical boundary conditions and centered. For simulations of energy minimization and molecular dynamics, Gromacs-4.5.5 software was used. The first part of the simulations occurred in vacuum, the other then in water. In order to keep the solution neutral, 24 molecules of water were randomly replaced by sodium ions. The system was minimized and equilibrated by series of restrained simulations (2.02 ns in total).

#### Simulation of SERCA in a phospholipid bilayer

In order to build a complex of SERCA protein with a phospholipid membrane, we used UCFS Chimera 1.10.2 software (University of California, San Francisco). The membrane composition was as follows: 10% phosphatidylserine, 30% phosphatidylethanolamine and 60% phosphatidylcholine. The membrane itself was created using CHARMM-GUI c40b1 software. Then, phospholipids at any distance greater than 0.35 nm from the enzyme were removed. The system was simulated in a periodic box of the sized of 15.65 × 15.65 × 18.00 nm^3^. The system was minimized and equilibrated by series of restrained simulations (3.02 ns in total).

### Biological assays

#### Cell lines and their cultivation

In this study, the following human cancer cell lines were used: LNCaP and PC-3 (prostate carcinoma), U-2 OS (osteosarcoma), MCF-7 (breast carcinoma), HT-29 (colon carcinoma), MIA PaCa-2 (pancreatic carcinoma), A549 (lung carcinoma); one transformed human cell line HEK 293T (embryonic kidney cells); one mouse cell line C2C12 (myoblasts) and a primary cell line MRC-5 (lung fibroblasts). The cell lines were purchased from American Type Culture Collection (ATCC, Manassas, USA) and from Sigma-Aldrich, USA. Unless otherwise specified, cells were cultured in medium (Thermo Fisher, USA) recommended by ATCC with stable glutamine dipeptide and supplemented with 10% fetal bovine serum (FBS; Thermo Fisher, USA). Cells were maintained at exponential phase of growth at 37 °C in humidified atmosphere with 5% CO_2_.

#### Cell viability assay

Viability of cells treated with the tested compounds was determined using WST-1 (Sigma-Aldrich, USA) assay by spectrophotometric detection (450 nm, reference 630 nm) as described in [10]. Briefly, WST-1 assay is based on reduction of a tetrazolium salt on soluble formazan in metabolically active cells. The measured absorbance is directly proportional to the number of metabolically active cells.

For this assay, cells were seeded into individual wells of 96-well plates (5000 cells per well) in 100 µL of cell culture medium supplemented with 10% FBS. The cells were incubated overnight (16 h) under standard cultivation conditions. Then, 100 µL of fresh media was added with the tested compounds (final concentration 0–50 µM) and, the cells were incubated for another 24, 48 and 72 h. Next, the medium was removed and the cells were incubated with 5 µL of WST-1 dissolved in 100 µL of high glucose DMEM without phenol red with 10% FBS for 2 h. Then, the absorbance was measured. Cells incubated with a vehicle (DMSO) in medium were used as control. All experiments were done in quadruplicates. The data were analyzed in Microsoft Excel; the deviations were calculated as standard error of the mean (SEM).

For primary peritoneal cells, a slightly modified procedure was used: the number of 1·10^5^ cells per well was cultivated overnight in triplicates, treated with the tested compounds and incubated for another 24 h. The amount of WST-1 was 10 µL per 100 µL of media and the incubation took 3 h. The results are expressed as the percentage of cytotoxicity relative to 100% of dead cells treated with 1% Triton X-100.

#### Cell uptake study

U-2 OS and MRC-5 cells (1·10^5^ cells per well) were seeded on 35 mm glass bottom dishes for live-cell imaging (MatTek Corporation, USA) and left to adhere for 24 h. Then, the cells were washed with phosphate-buffered saline (PBS) and incubated with compound **5** or dansyl amide fluorophore (0.1–2.0 µM) dissolved in FluoroBrite DMEM medium (Thermo Fisher, USA) for 0.5–2.5 h. After the incubation period, the cells were washed with PBS and fresh FluoroBrite DMEM was added.

#### Determination of localization in cell organelles

U-2 OS and MRC-5 cells were seeded and influenced by the tested compounds as described in section “Cell uptake study”. To assess the intracellular localization of compound **5** in MRC-5 cells, an endoplasmic reticulum marker ER-Tracker™ Red (120 nM, 30 min; ThermoFisher Scientific, USA) and a mitochondria-specific dye (70 nM, 30 min; UCT Prague, CZ) from [20] were used. In U-2 OS cells, the endoplasmic reticulum was visualized by transfection with a plasmid DNA coding mCherry-ER (0.5 µg) using Fugene HD (Promega, USA; DNA/Fugene HD = 1:3).

#### Fluorescence microscopy

The intracellular localization of compound **5** was studied by real-time live-cell fluorescence microscopy using an inverse fluorescence microscope Olympus IX-81 operated by xCellence software (Olympus, Japan) and equipped with a high-stability 150 W xenon arc burner and EM-CCD camera C9100-02 (Hamamatsu, Germany). A 60× oil immersion objective (Olympus, Japan) with a numerical aperture of 1.4 was used. All images were deconvolved and background-corrected by xCellence software.

#### Animals and cells

For isolation of resident peritoneal macrophages, female Wistar rats weighing 175–185 g (VELAZ, Czech Republic) were used. They were kept in plastic cages under standard conditions, i.e., a temperature of 22 ± 2 °C, 12/12 h of light/dark cycle, relative humidity (50 ± 10%) and standard pelleted diet and water were provided ad libitum. Procedures were approved by Institutional protocol MSMT-15894/2013-310. Rats were sacrificed by cervical dislocation. The resident peritoneal macrophages of individual rats were collected by a lavage using sterile saline. The cells were washed, resuspended and seeded into 96-well round-bottom microplates (2·10^5^ cells per well) in complete RPMI-1640 medium containing 10% heat-inactivated FBS, 2 mM ʟ-glutamine, 50 µg·mL^−1^ gentamicin, and 5·10^−5^ M 2-mercaptoethanol (all Sigma-Aldrich, St. Louis, MO, USA). The treated or untreated cells (DMSO as a vehicle) were cultured for 24 h and plates were maintained at 37 °C, 5% CO_2_, in a humidified Heraeus incubator. Stock solutions of compound **1** and **5** were prepared 100 mM in DMSO. For the cultivation of macrophages and assessment of toxicity and NO level, the majority of chemicals was purchased from Sigma-Aldrich and Merck-Sigma, USA.

#### Nitrite oxide production in primary rat macrophages

To evaluate immunomodulatory activity of compound **1** and its derivatives, 100 pg·mL^−1^ of lipopolysaccharide (LPS) was applied to cells in appropriate wells. After 24 h, the concentration of nitrites was detected in individual supernatants (50 µL) incubated for 10 min at ambient temperature with an aliquot of the Griess reagent (1% sulphanilamide/0.1% naphthylendiamine/2.5% H_3_PO_4_). The absorbance was recorded at 540 nm using a microplate spectrophotometer (Tecan, Austria). A nitrite calibration curve was used to convert absorbance to concentration (µM) of nitrite.

#### Cytokine assay

Analogous to NO detection, supernatants of all samples were analyzed for cytokine content after 24 h. The concentration of secreted TNF-α was determined by ELISA assay (R&D Systems, Abingdon, UK) following manufacturer protocol.

#### Statistical analysis

Analysis of variance (ANOVA) and graphical presentation of data were done using GraphPad Prism 6.05, San Diego, CA.

### Abbreviations

4-DMAP, 4-(*N*,*N*-dimethylamino)pyridine; AcOEt, ethyl acetate; DCC, dicyclohexylcarbodiimide; DCU, dicyclohexylurea; Dns, dansyl amide; DMAP, 4-dimethylaminopyridine; DTB, 8-*O*-(dodecanoyl-8-*O*-debutanoyltrilobolid); IL-6, interleukin 6; IL-1β, interleukin 1β; INF-γ, interferon gamma; TBTA, tris[(1-benzyl-1*H*-1,2,3-triazol-4-yl)methyl]amine; TEA, triethylamine; THF, tetrahydrofuran; TLC, thin-layer chromatography; TNF-α, tumor necrosis factor alpha; SCE, supercritical CO_2_ extraction; SL, sesquiterpene lactones.

### Comment on nomenclature

This article uses semitrivial terpene nomenclature and hence the numbering of compounds atoms may differ from these obtained by using IUPAC names.

## Supporting Information

File 1 Additional experimental data.
