# Supplementary material for: Archangelolide: A sesquiterpene lactone with immunobiological potential from Laserpitium archangelica
Source: Beilstein J Org Chem. 2019 Aug 13;15:1933–44. doi: 10.3762/bjoc.15.189 (PMC6720059; doi:10.3762/bjoc.15.189)
Supplement: File 1 — Additional experimental data. [file Beilstein_J_Org_Chem-15-1933-s001.pdf]

BEILSTEIN JOURNAL OF ORGANIC CHEMISTRY

Supporting Information

for

Archangelolide: A sesquiterpene lactone with immunobiological potential from *Laserpitium archangelica*

Silvie Rimpelová, Michal Jurášek, Lucie Peterková, Jiří Bejček, Vojtěch Spiwok, Miloš Majdl, Michal Jirásko, Miloš Buděšínský, Juraj Harmatha, Eva Kmoníčková, Pavel Drašar and Tomáš Ruml

Beilstein J. Org. Chem. **2019,** *15,* 1933–1944. doi:10.3762/bjoc.15.189

Additional experimental data

License and Terms: This is a supporting information file under the terms of the Creative Commons Attribution License (http://creativecommons.org/licenses/by/4.0). Please note that the reuse, redistribution and reproduction in particular requires that the authors and source are credited.
The license is subject to the *Beilstein Journal of Organic Chemistry* terms and conditions: (https://www.beilstein-journals.org/bjoc)

1 General techniques and apparatus

For thin layer chromatography (TLC), we used plates coated by silica gel bound with starch for detection in UV light (TLC Silica gel 60 F254, Merck). For visualization, diluted sulfuric acid in MeOH was used and the plates were then heated. For column chromatography, silica gel (30–60 μm, SiliTech, MP Biomedicals) was used.

HRMS data were measured by Micro Q-TOF with ESI ionization. FTIR spectra were measured with a Nicolet iS10 using the ATR technique using KBr crystals. Specific rotations were measured by using a Autopol VI polarimeter. For thin layer chromatograms (TLC), we used plates coated with a silica gel bound to starch for detection in UV light (TLC Silica gel 60 F254, Merck).

The NMR spectra were measured with a Bruker AVANCE-600 instrument (^{1}H at 600.13 MHz and ^{13}C at 150.9 MHz) with a cryo-probe in $CDCl_3$ at 25 °C. Structural assignments of proton and carbon signals were achieved by combining one-dimensional ^{1}H and ^{13}C spectra with two-dimensional homonuclear 2D-H,H-COSY, 2D-H,H-ROESY and heteronuclear 2D-H,C-HSQC and 2D-H,C-HMBC spectra. ^{13}C and ^{1}H NMR data of compounds **1**, **3**, **4** and **5** are summarized in Table S1. For column chromatography, silica gel (30–60 μm, SiliTech, MP Biomedicals) was used. The click reactions were carried out in a microwave reactor Biotage Initiator Classic.

Table S1: ^{13}C and ^{1}H NMR data of compounds **1**, **3**, **4** and **5** in $CDCl_3$. Data for a substituent in the position 11 in compounds **4** and **5** are shown in Figure S1.

**Position**	**1**		**3**		**4**		**5**	
	**^{13}C**	**^{1}H**	**^{13}C**	**^{1}H**	**^{13}C**	**^{1}H**	**^{13}C**	**^{1}H**
1	52.70	3.32 dd	52.94	3.29 dd	52.65	3.32 dd	52.69	3.33 dd
2	79.05	5.82 m	78.63	5.81 m	79.04	5.83 m	79.03	5.83 m
3	127.17	5.66 m	126.96	5.65 m	127.16	5.66 m	127.23	5.66 m
4	148.94	--	148.77	--	148.86	--	148.86	--
5	50.13	3.05 m	49.30	3.07 dd	50.10	3.05 dd	50.12	3.05 dd
6	76.82	4.81 dd	77.30	4.66 dd	76.85	4.82 dd	77.06	4.84 dd
7	48.15	3.61 dd	53.25	3.06 dd	48.22	3.60 dd	48.25	3.61 dd
8	65.43	5.61 td	64.74	5.69 td	65.50	5.60 td	65.49	5.60 td
9	44.22	2.59 dd	43.59	2.58 dd	44.19	2.60 dd	44.19	2.58 dd
		2.06 dd		2.14 dd		2.07 dd		2.07 dd
10	80.87	--	81.10	--	80.83	--	80.87	--
11	78.16	--	73.22	--	78.24	--	78.50	--
12	173.60	--	178.51	--	173.56	--	173.73	--
13	20.43	1.58 s	22.40	1.57 s	20.34	1.57 s	20.40	1.57 s
14	26.28	1.43 s	26.44	1.44 s	26.19	1.44 s	26.27	1.43 s
15	17.72	1.94 br q	17.43	1.92 br q	17.67	1.94 br q	17.70	1.94 br q
2-OAng	167.46	--	167.41	--	167.41	--	167.45	--
	127.79	--	127.62	--	127.74	--	127.78	--
	137.86	6.04 qq	137.94	6.05 qq	137.80	6.04 qq	137.85	6.04 qq
	15.73	1.95 dq	15.63	1.95 dq	15.68	1.95 dq	15.72	1.95 dq
	20.54	1.86 p	20.41	1.86 p	20.49	1.86 p	20.52	1.86 p
8-OMeBu	174.85	--	175.18	--	174.83	--	174.82	--
	41.35	2.31 m	41.05	2.35 m	41.28	2.33 m	41.30	2.29 m
	26.42	1.72 m	26.23	1.72 m	26.34	1.72 m	26.40	1.68 m
		1.46 m		1.45 m		1.46 m		1.44 m
	11.68	0.93 t	11.48	0.91 t	11.66	0.93 t	11.69	0.91 t
	16.36	1.15 d	16.23	1.14 d	16.37	1.15 d	16.40	1.12 d
10-OAc	170.07	--	170.09	--	170.04	--	170.09	--
	22.35	2.02 s	22.25	2.05 s	22.30	2.02 s	22.33	2.02 s
11-OAc	169.77	--	--	--	--	--	--	--
	20.97	2.08 s	--	--	--	--	--	--
11-OH	--	--	--	3.01 br s	--	--	--	--

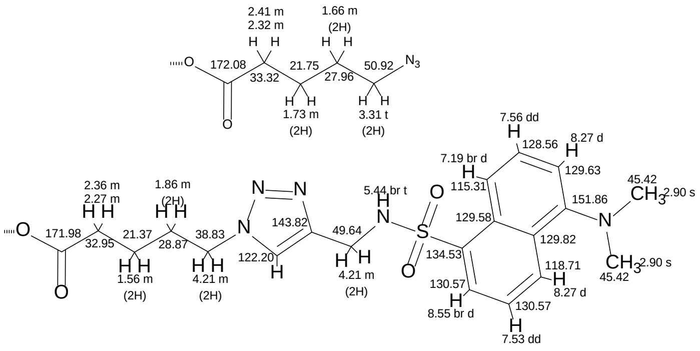

Figure S1: Assignments of the ^{1}H and ^{13}C signals for the azidopentanoate and "dansylated" residues.

2 IR spectroscopy, optical rotations

***Archangelolide* (1):** α_D^{20} **= −**117 (measured with *c*=1, $CHCl_3$) IR: 3455 (*vw*), 2969 (*m*), 2931 (*m*), 2877 (*w*), 1794 (γ-lactone, *vs*), 1736 (*vs*), 1694 (*w*), 1456 (*m*), 1381 (*m*), 1370 (*m*), 1238 (*vs*), 1152 (γ-lactone, *vs*), 1099 (*s*), 1042 (*m*), 1018 (*s*), 980 (*m*).

For isolation of archangelolide, fine ground seeds of *L. archangelica* were used (Figure S2).

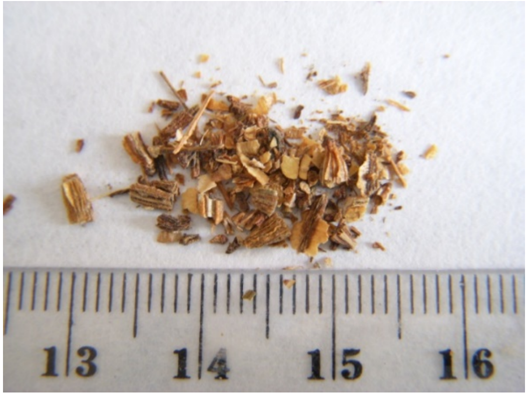

Figure S2: Ground seeds of *Laserpitium archengelica*, Wulfen.

***11-Deacetyl archangelolide* (3):** α_D^{20} **= −**135.2 (measured with *c*=0.25, DCM). IR: 3462 (*br. m*), 2971 (*m*), 2934 (*m*), 2877 (*w*), 2254 (*vw*), 1785 (γ-lactone, *s*), 1732 (*vs*), 1649 (*w*), 1457 (*m*), 1379 (*m*), 1304 (*w*), 1234 (*vs*), 1155 (γ-lactone, *vs*), 1107 (*m*), 1084 (*m*), 1042 (*m*), 1014 (*m*), 981 (*m*).

***11-Azidovaleroyl archangelolide* (4):** α_D^{20} **=** −201.2 (measured with *c*=0.25, DCM). IR: 2968 (*m*), 2934 (*m*), 2876 (*w*-*m*), 2097 (ν N_3, *s*), 1792 (γ-lactone, *s*), 1734 (*vs*), 1649 (*w*), 1456 (*m*), 1381 (*m*), 1351 (*m*), 1236 (*s*), 1152 (γ-lactone, *s*), 1099 (*m*), 1042 (*m*), 1014 (*m*).

***11-(5-(4-((5-(Dimethylamino)naphthalene-1-sulfonamido)methyl)-1H-1,2,3-triazol-1-yl)pentanoyl archangelolide* (5):** α_D^{20} **=** −72.8 (measured with *c*=0.25, DCM). IR: 3287 (*w*), 2958 (*s*), 2924 (*vs*), 2853 (*s*), 1789 (γ-lactone, *s*), 1733 (*vs*), 1456 (*m*), 1382 (*m*), 1321 (*m*), 1258 (*s*), 1236 (*s*), 1145 (γ-lactone, *vs*), 1099 (*s*), 1043 (*s*), 1018 (*s*).

3 HRMS and NMR analyses of the compounds

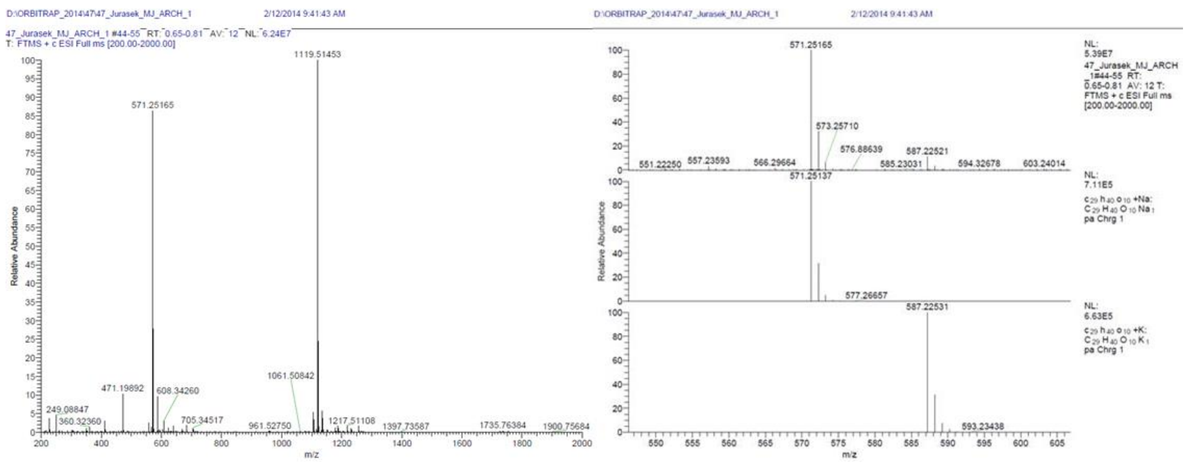

Figure S3: HRMS of archangelolide, compound **1**. HRMS-ESI: *monoisotopic mass* 548.26215 Da, found (*m*/*z*) 571.25165 corresponds to $[M+Na]^+$, 587.22521 to $[M+K]^+$ and 1119.51453 to $[2M+Na]^+$ ion in the proposed structure.

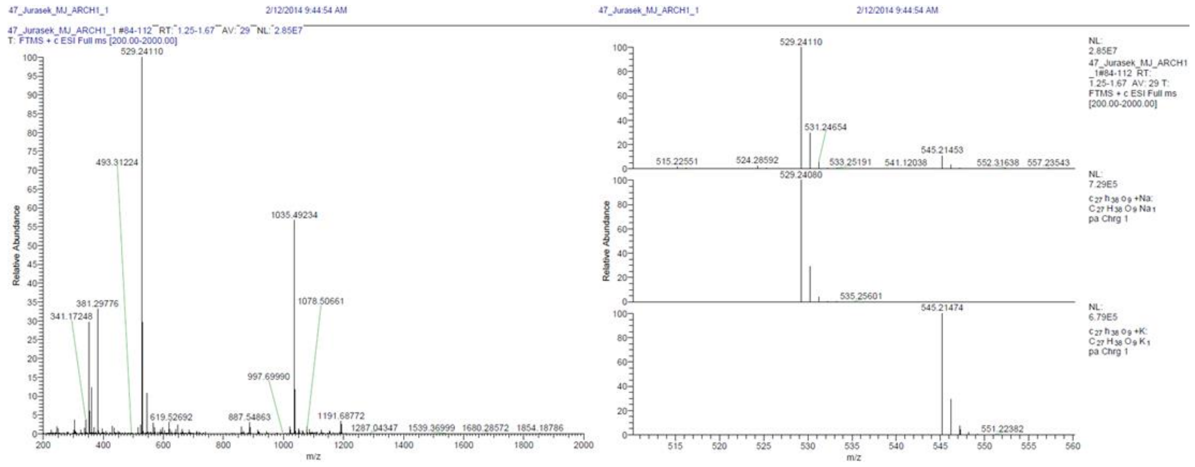

Figure S4: HRMS of compound **3**. HRMS-ESI: *monoisotopic mass* 506.25158 Da, found (*m*/*z*) 529.24110 corresponds to $[M+Na]^+$, 545.21453 to $[M+K]^+$ and 1035.49234 to $[2M+Na]^+$ ion in the proposed structure.

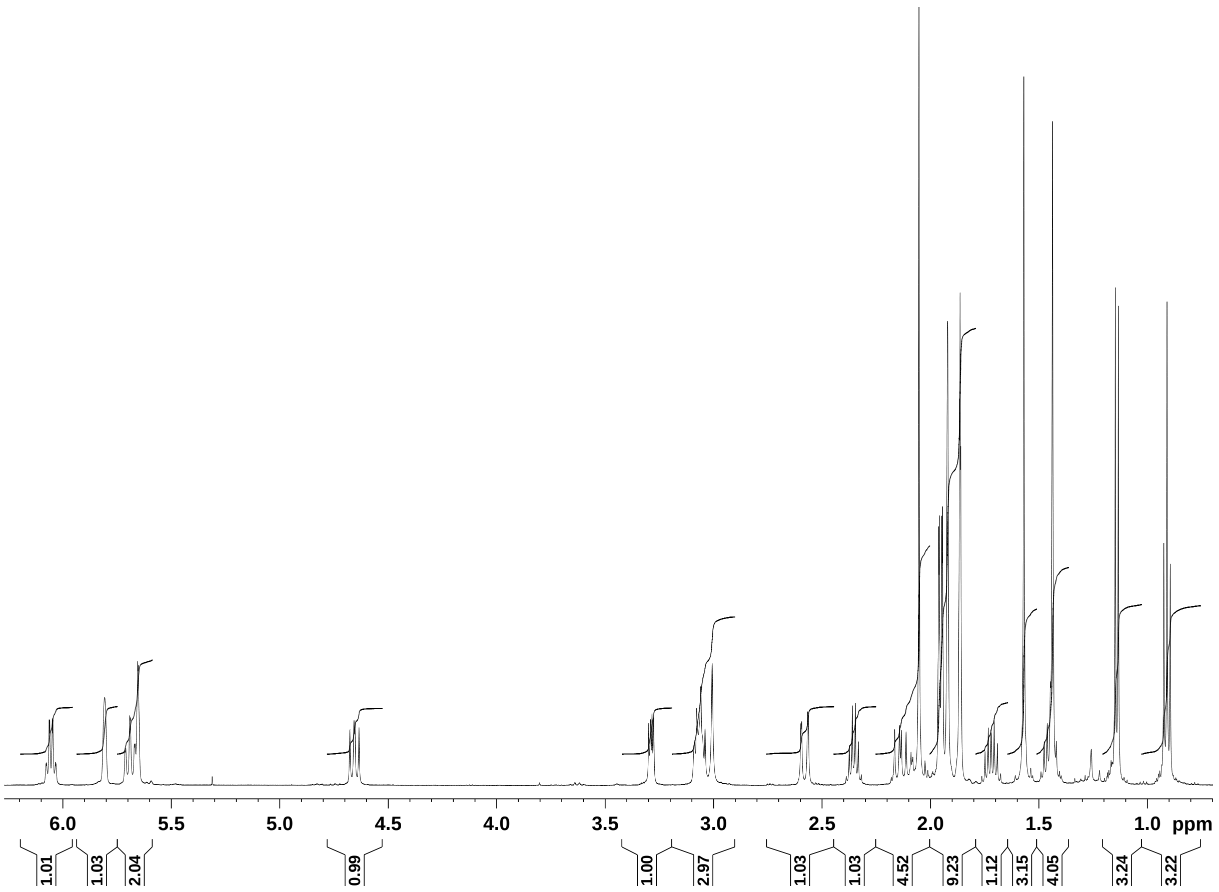

Figure S5: ^{1}H NMR spectrum (500 MHz) of compound **3** in $CDCl_3$.

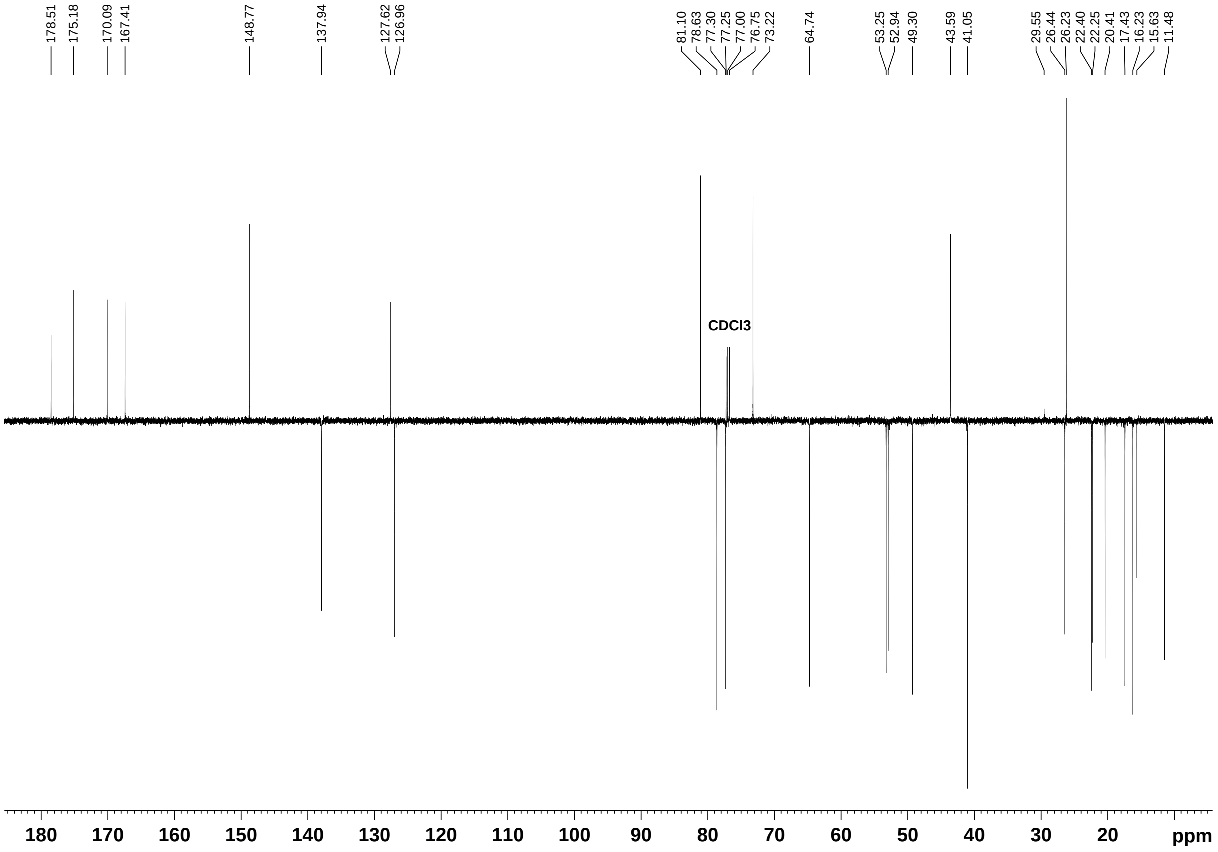

Figure S6: ^{13}C-APT NMR spectrum (125.7 MHz) of compound **3** in $CDCl_3$.

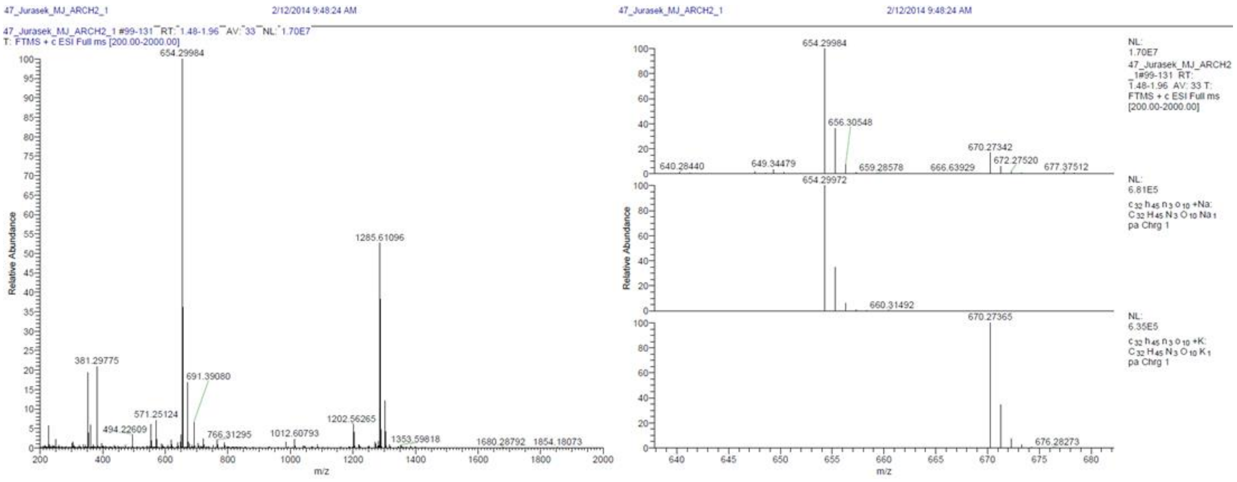

Figure S7: HRMS of compound **4**. HRMS-ESI: *monoisotopic mass* 631.31049 Da, found (*m*/*z*) 654.29984 corresponds to $[M+Na]^+$, 670.27342 to $[M+K]^+$ and 1285.61096 to $[2M+Na]^+$ ion in the proposed structure.

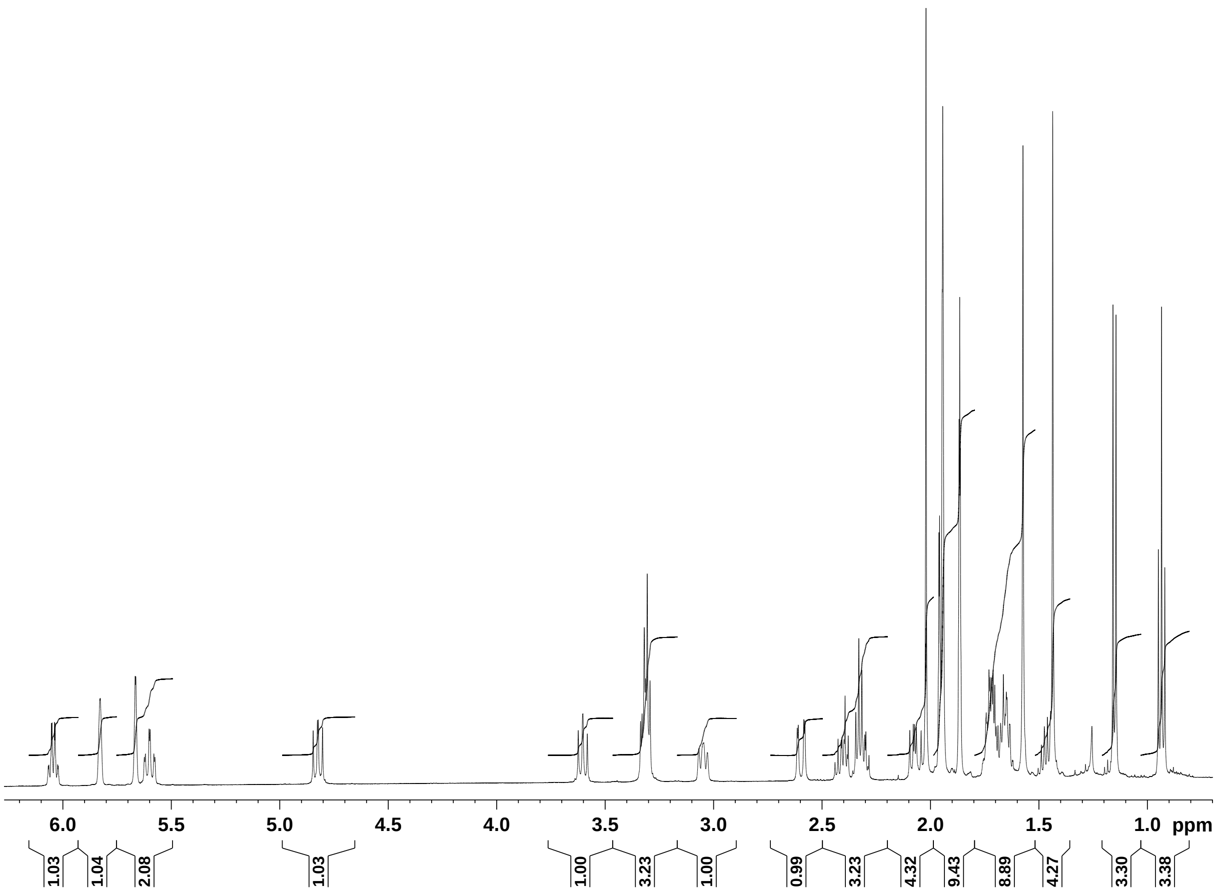

Figure S8: ^{1}H NMR spectrum (500 MHz) of compound **4** in $CDCl_3$.

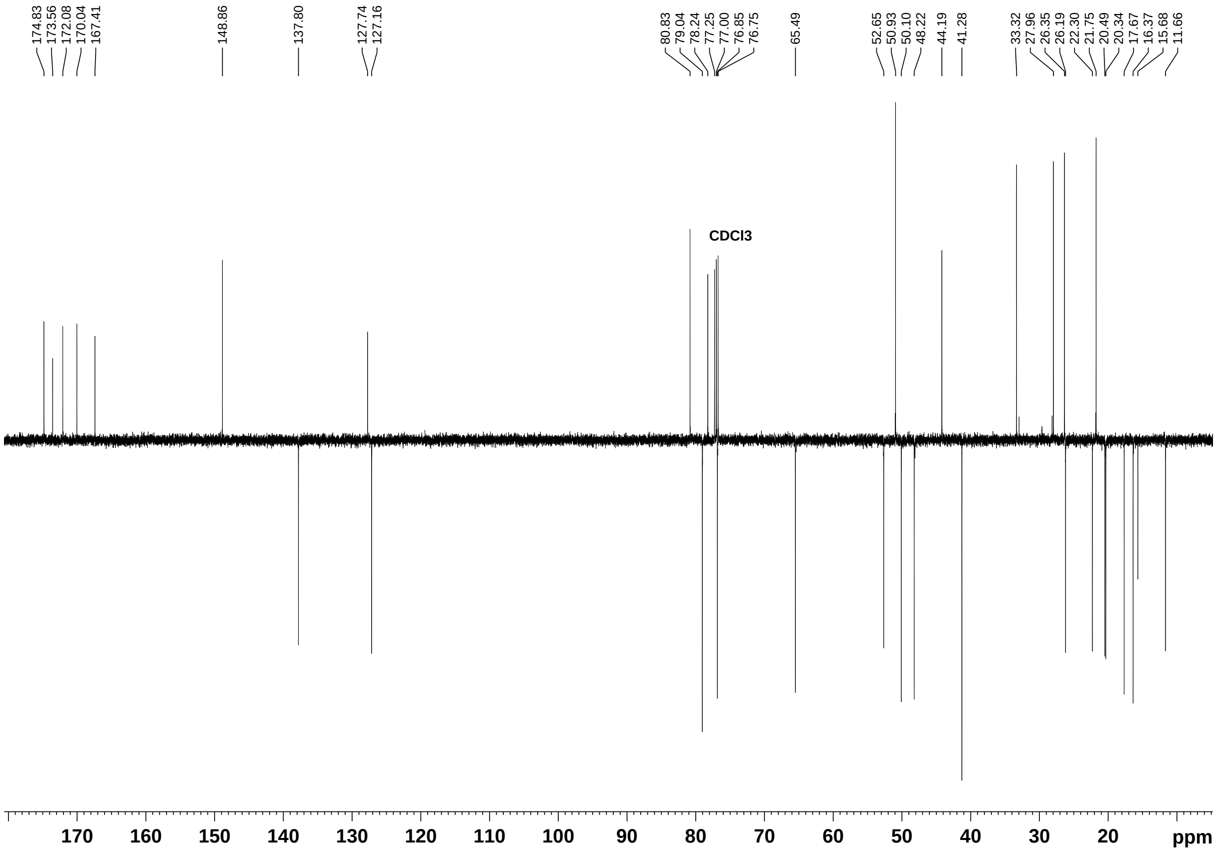

Figure S9: ^{13}C-APT NMR spectrum (125.7 MHz) of compound **4** in $CDCl_3$.

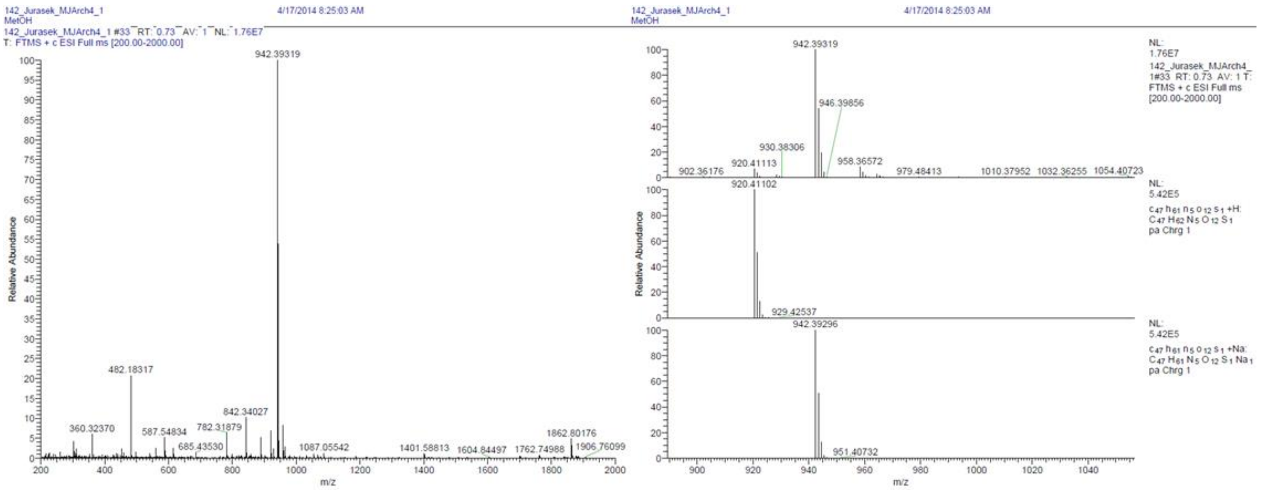

Figure S10: HRMS of compound **5**. HRMS-ESI: *monoisotopic mass* 919.40374 Da, found (*m*/*z*) 920.39319 corresponds to $[M+H]^+$, 942.39319 to $[M+Na]^+$ ion in the proposed structure.

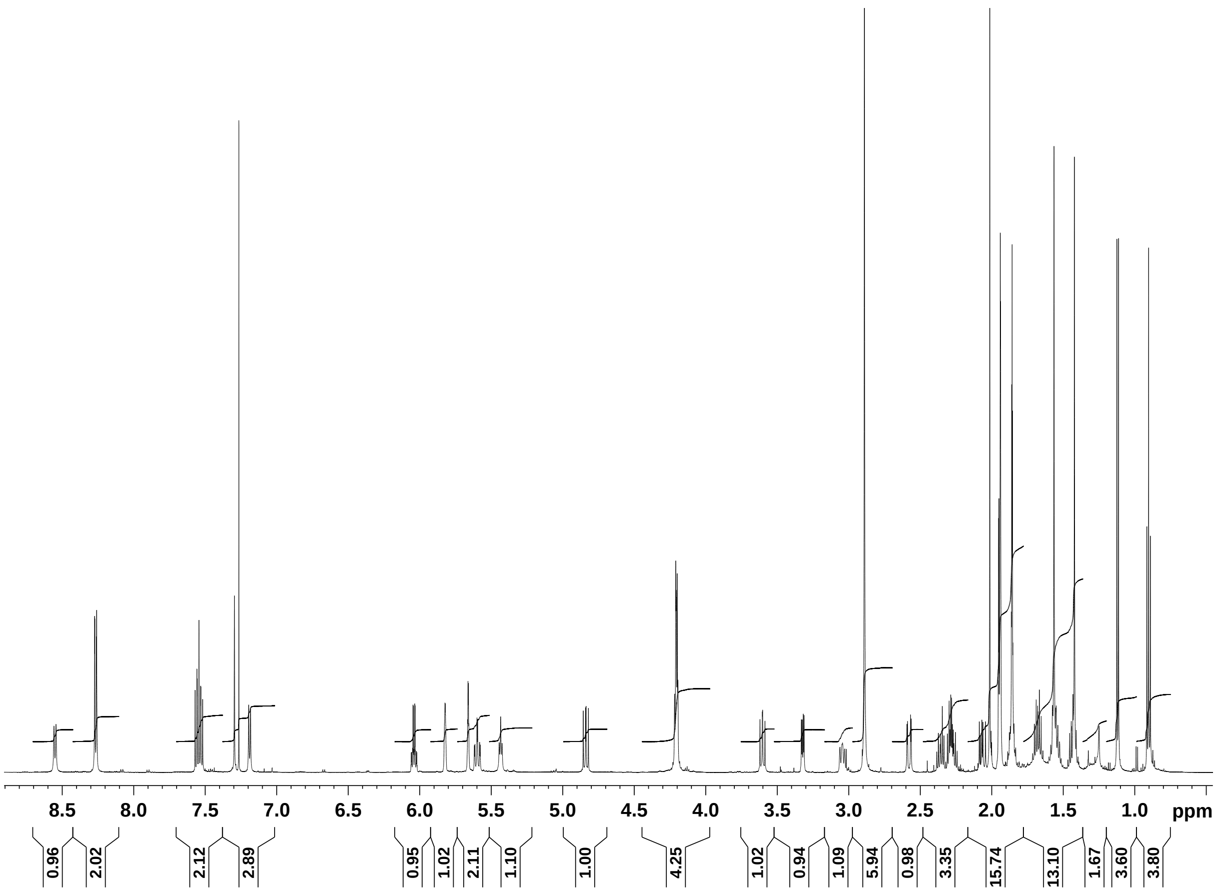

Figure S11: ^{1}H NMR spectrum (600 MHz) of compound **5** in $CDCl_3$.

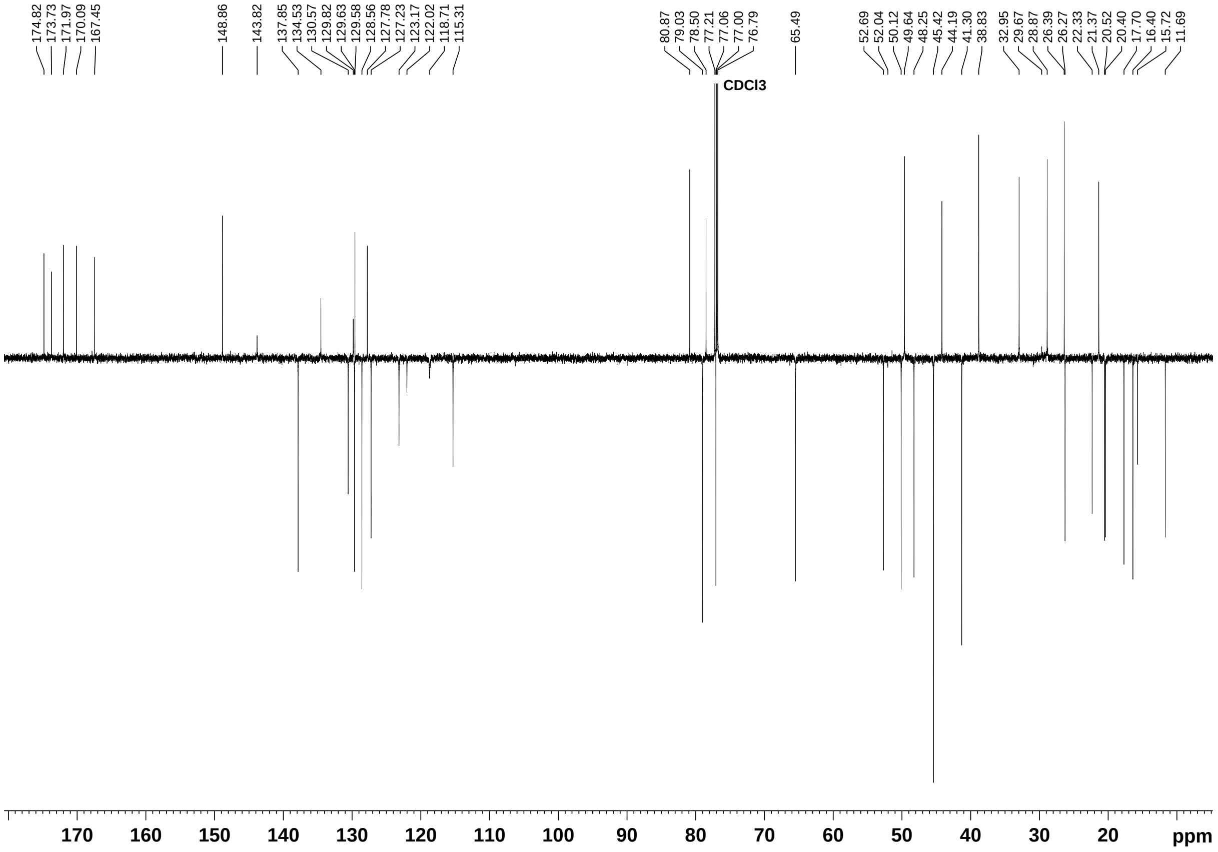

Figure S12: ^{13}C-APT NMR spectrum (150.9 MHz) of compound **5** in $CDCl_3$.

4 HPLC Analyses of the tested compounds

HPLC analyses were performed using C18 column (0.5 × 280 mm) with UV detection. We used gradient elution with a flow rate of 0.8 mL·min^{-1} with the following systems: system A – 100 % water and system B 100 % MeOH. The method was optimized for 15 min with the following gradient:

	Time [min]	B [%]
1	*0*	*40*
2	*5*	*100*
3	*15*	*100*

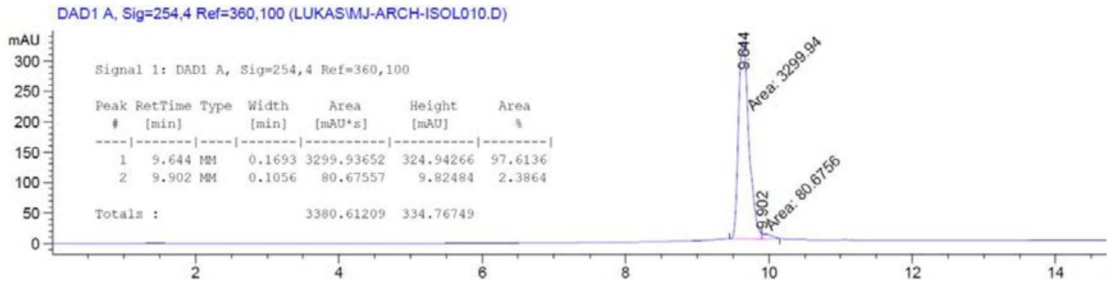

Figure S13: HPLC of compound **1**.

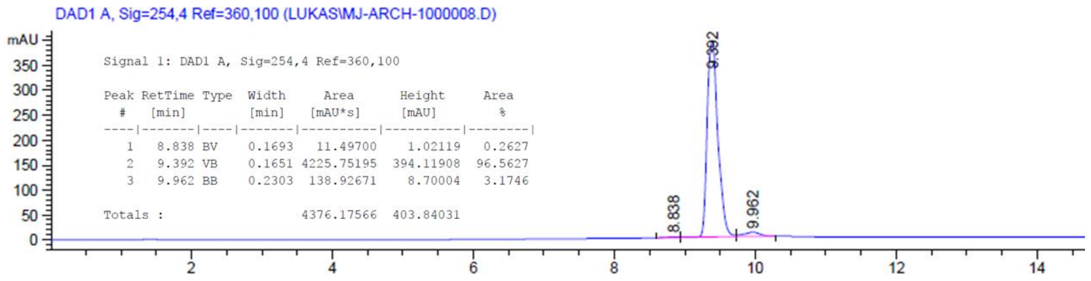

Figure S14: HPLC of compound **3**.

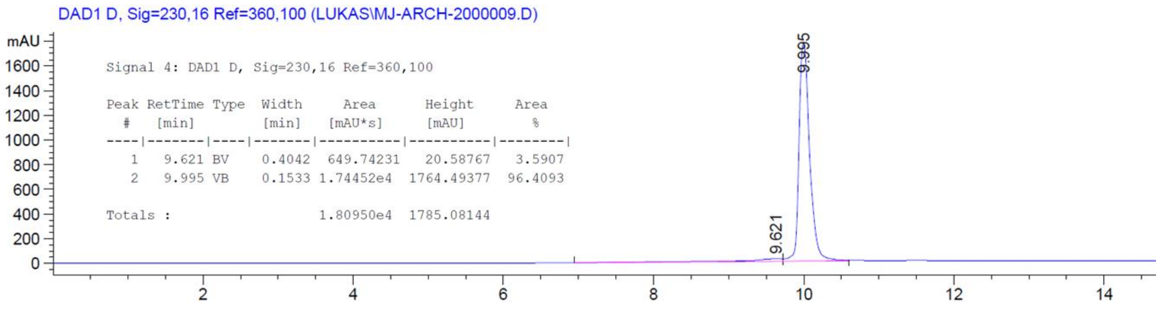

Figure S15: HPLC of compound **4**.

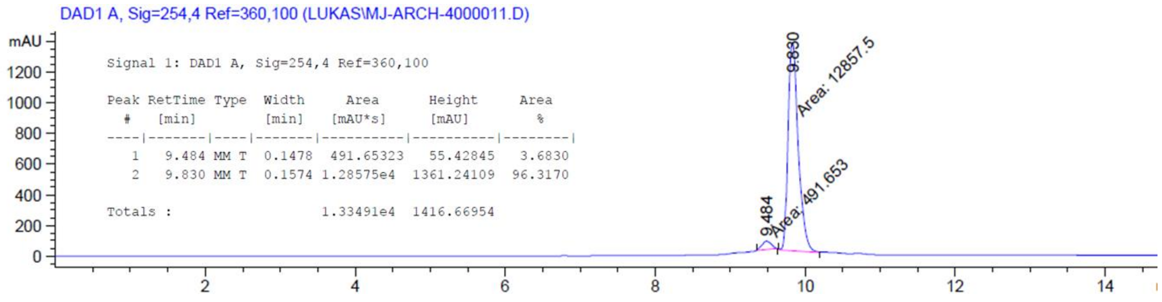

Figure S16: HPLC of compound **5**.

5 Fluorescence properties of compound 5

The fluorescence spectra were recorded using a Cary Eclipse fluorescence spectrometer. The sample was dissolved in MeOH to a 10^{-4} M solution, which was further diluted with MeOH or 0.01 M phosphate-buffered saline (PBS) to 10^{-5} M. The PBS sample contained 10% of MeOH. An excitation wavelength of 340 nm was used.

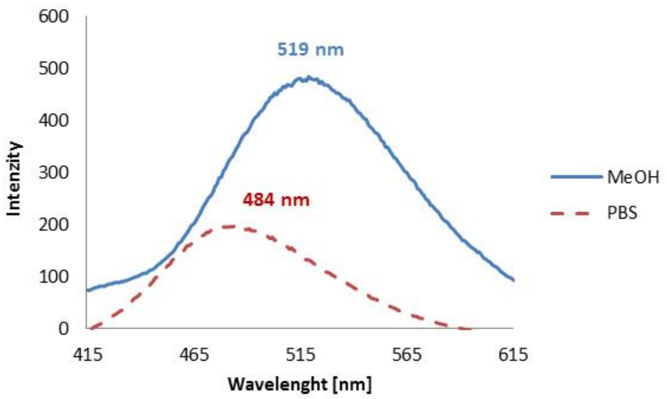

Figure S17: Fluorescence properties of fluorescent archangelolide conjugate (compound **5**). Methanol (blue line) and PBS (red dashed line).

6 Intracellular localization

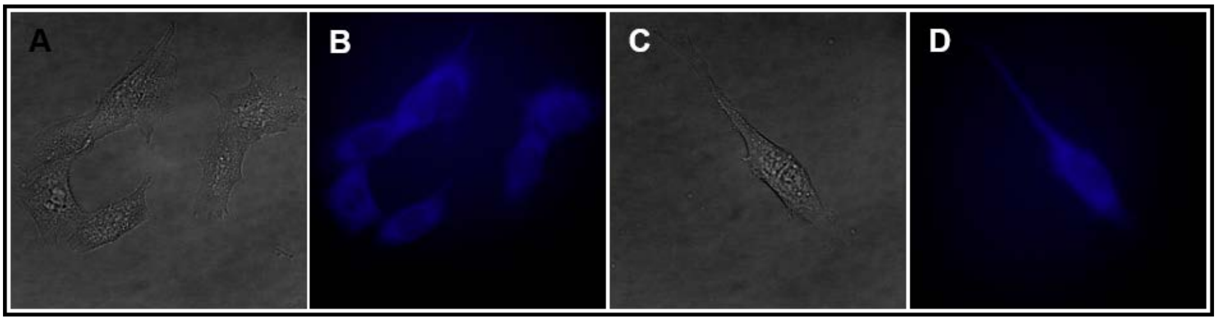

Figure S18: Intracellular localization of dansyl amide in human cells from osteosarcoma (U-2 OS). A, C) Bright-field images; B, D) fluorescence microscopy of living cells treated with 2 μM concentration of dansyl amide for 2 h.

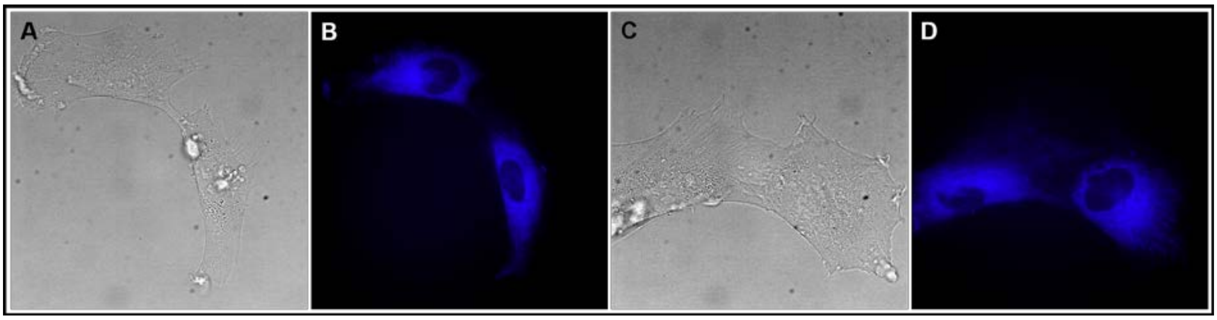

Figure S19: Intracellular localization of archangelolide-dansyl (**5**) in primary cells of lung fibroblast (MRC-5). A, C) Bright-field images; B, D) fluorescence microscopy of living cells treated with 1 μM concentration of compound **5** for 2 h.

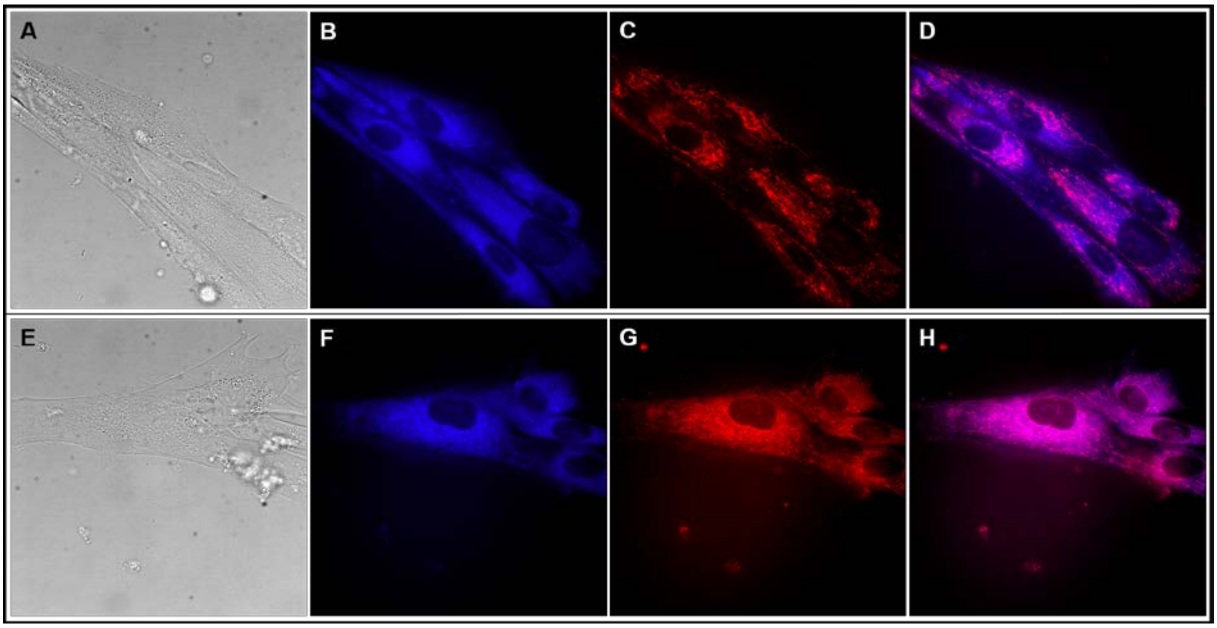

Figure S20: Co-localization of archangelolide-dansyl (**5**) with a marker of endoplasmic reticulum (top row) and with a mitochondrial marker (bottom row) in primary cells of lung fibroblast (MRC-5). A, E) Bright-field images. Fluorescence microscopy of living cells treated with 1 μM concentration of compound **5** for 2 h (images B and F) and a mitochondria-specific dye from [1] (10 min; image C) or ER-Tracker Red (30 min; image G). D, H) merged images.

7 Cytotoxicity

Table S2: Viability of cells treated with archangelolide (compound **1**) for 24, 48 and 72 h measured by WST-1 assay. The IC_{50} value represents the inhibitory concentration of a compound necessary to kill 50% of cells.

**Comp.**		**IC_{50} [μM]**				
	**Time**	**MRC-5**	**LNCaP**	**PC-3**	**U-2 OS**	**MCF-7**
1	**24 h**	n.r.	n.r.	n.r.	n.r.	n.r.
	**48 h**	n.r.	n.r.	n.r.	n.r.	n.r.
	**72 h**	n.r.	48.6	n.r.	n.r.	n.r.

Table S3: Viability of cells treated with archangelolide and its derivatives for 24, 48 and 72 h measured by WST-1 assay. The IC_{50} value represents the inhibitory concentration of a compound necessary to kill 50% of cells.

**Comp.**	**IC_{50} [μM]**														
	**HT-29**			**C2C12**			**MiaPaCa-2**			**HEK 293T**			**A549**		
**Time**	**24 h**	**48 h**	**72 h**	**24 h**	**48 h**	**72 h**	**24 h**	**48 h**	**72 h**	**24 h**	**48 h**	**72 h**	**24 h**	**48 h**	**72 h**
**1**	n.r.[a]	n.r.	n.r.	n.r.	n.r.	n.r.	n.r.	n.r.	n.r.	n.r.	n.r.	42.3	n.r.	n.r.	n.r.
**3**	n.r.	n.r.	n.r.	n.r.	n.r.	n.r.	n.r.	n.r.	n.r.	n.r.	n.r.	n.r.	n.r.	n.r.	n.r.
**4**	n.r.	n.r.	n.r.	n.r.	n.r.	n.r.	n.r.	n.r.	n.r.	n.r.	n.r.	37.5	n.r.	n.r.	n.r.
**5**	n.r.	n.r.	n.r.	n.r.	n.r.	n.r.	n.r.	n.r.	n.r.	n.r.	n.r.	n.r.	n.r.	n.r.	n.r.
**Dns**	n.r.	n.r.	n.r.	n.r.	n.r.	n.r.	n.r.	n.r.	n.r.	n.r.	n.r.	n.r.	n.r.	n.r.	n.r.

[a]n.r. – not reached

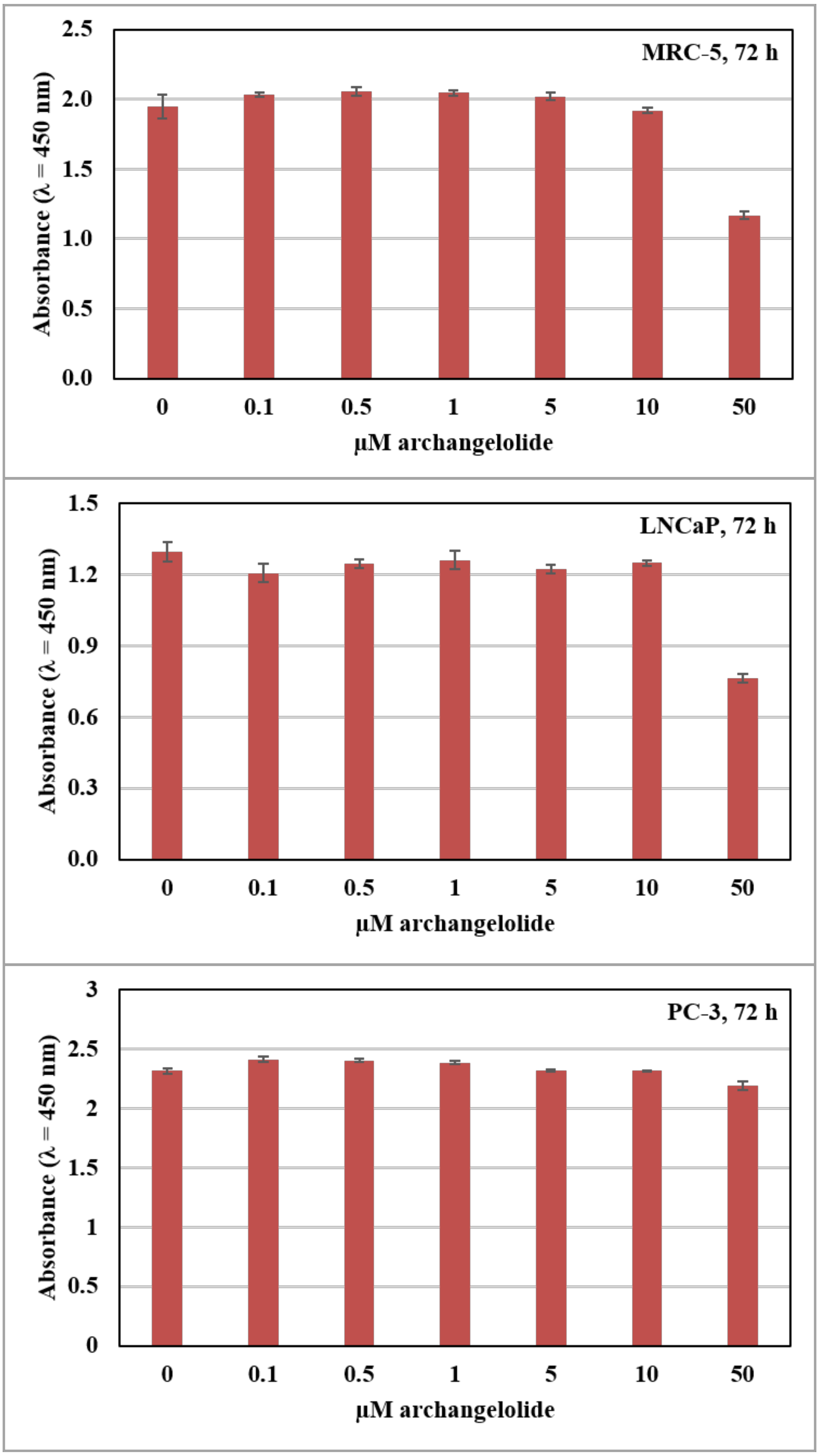

Figure S21: Viability of primary fibroblasts (MRC-5) and cells derived from prostate carcinomas (LNCaP, PC-3) treated with archangelolide for 72 h measured by WST-1 assay.

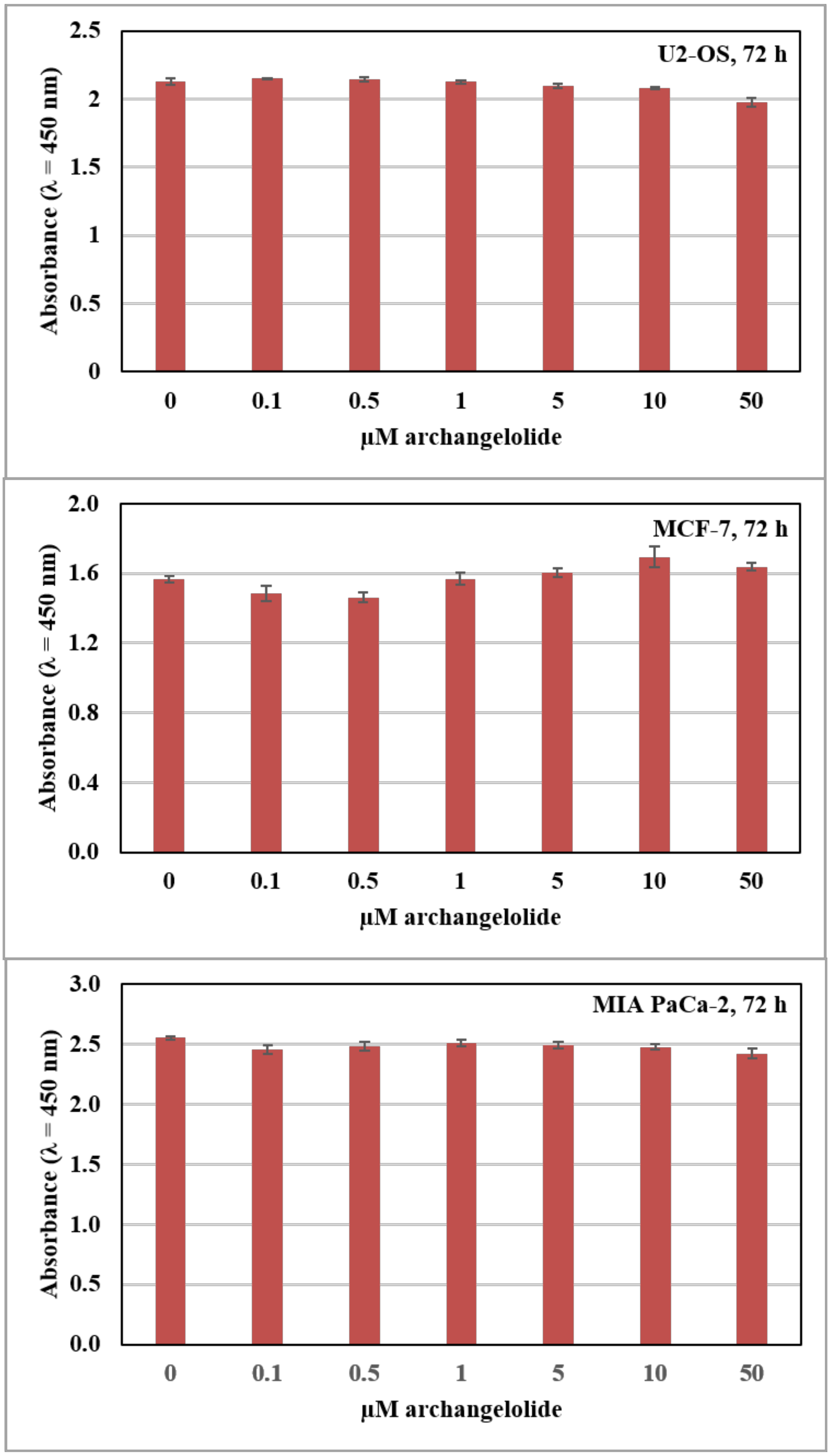

Figure S22: Viability of cells from human osteosarcoma (U-2 OS), breast carcinoma (MCF-7) and pancreas adenocarcinoma (Mia PaCa-2) treated with archangelolide for 72 h measured by WST-1 assay.

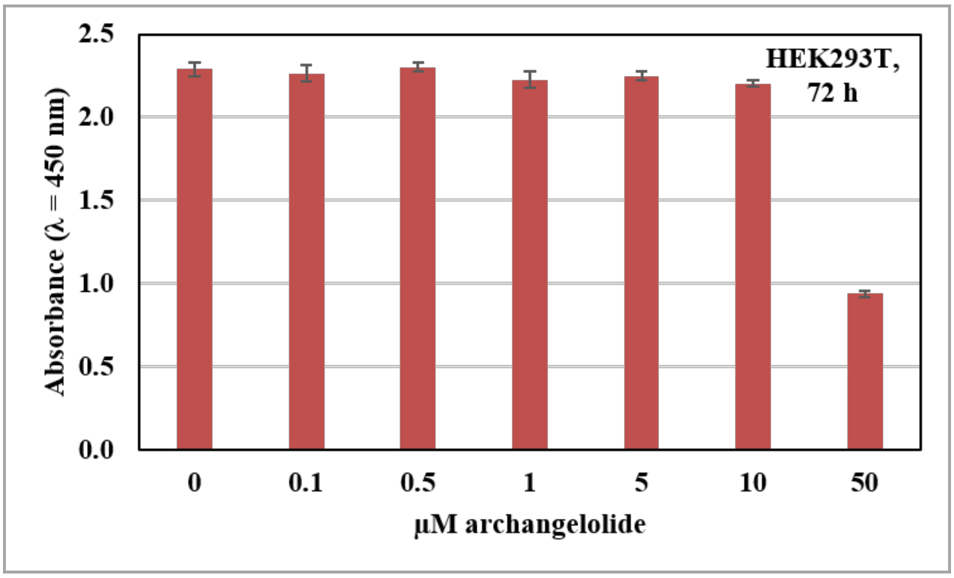

Figure S23: Viability of human embryonic fibroblasts from kidney (HEK 293T) treated with archangelolide for 72 h measured by WST-1 assay.

Table S4: Hydrogen bonding and hydrophobicity of SLs.

	thapsigargin	trilobolide	archangelolide
formula	$C_{34}H_{50}O_{12}$	$C_{27}H_{38}O_{10}$	$C_{29}H_{40}O_{10}$
log$P^{a,b}$	6.98	4.26	4.9
number of hydrogen bond donor atoms[b]	2	2	0
number of hydrogen bond acceptor atoms[b]	12	10	10

[a] octanol–water partition coefficient

[b] predicted with: Advanced Chemistry Development, Toronto, ACD/Percepta 14.2.0 (Build 2977)

8 Super critical extraction method – why did we use it?

There are multiple advantages to supercritical CO_2 extraction over extraction with organic solvents and the procedure is extensively used in extraction of natural compounds [2]. The diffusivity is increased in supercritical CO_2 compared to solvent extraction and consequently the duration of the process is reduced [3]. Supercritical CO_2 is a good solvent for the rather non-polar compounds we were extracting and was used in the past to extract other SLs, such as parthenolide [4-6]. Furthermore, the extraction can be finetuned by varying pressure as well as

temperature, which is favorable for thermally unstable compounds and particularly natural products. Also, the use of organic solvent is significantly reduced during supercritical CO_2 extraction compared to conventional solvent extraction [3]. Finally, a slight disadvantage of this procedure may come forward when plant material rich in water is used for extraction [3]. However, this was not the case here, as we used seeds, which generally have low water content. We admit that we may have not used the method to its full potential. As we know from the literature, the procedure could be optimized to enrich for the desired compound [3], which we did not attempt. However, this does not in any way diminish the fact that supercritical CO_2 extraction is a facile and rapid method by means of which we reached our objective, that is, the isolation of archangelolide.

References

[1] Rimpelová, S.; Bříza, T.; Králová, J.; Záruba, K.; Kejík, Z.; Císařová, I.; Martásek, P.; Ruml, T.; Král, V. *Bioconjug. Chem.* **2013,** *24*, 1445-1454. doi:10.1021/bc400291f

[2] Reverchon, E.; De Marco, I. *J. Supercrit. Fluids* **2006**, *38*, 146–166. doi:10.1016/j.supflu.2006.03.020

[3] Lang, Q.; Wai, C. *Talanta* **2001**, *53*, 771–782. doi:10.1016/S0039-9140(00)00557-9

[4] Castaneda-Acosta, J.; Cain A. W.; Fisher, N. H.; Knopf F. C. *J. Agric. Food Chem.* **1995**, *43*, 63–68. doi:10.1021/jf00049a013

[5] Kery, A.; Ronyai, E.; Simandi, B.; Lemberkovics, E.; Keve, T.; Deak, A.; Kemeny, S. *Chromatographia* **1999**, *49*, 503–508. doi:10.1007/BF02467749

[6] Smith, R. M.; Burford M. D. *J. Chromat. A* **1992**, *627*, 255–261. doi:10.1016/0021-9673(92)87205-M
